# VCAM1 acts in parallel with CD69 and is required for the initiation of oligodendrocyte myelination

**DOI:** 10.1038/ncomms13478

**Published:** 2016-11-23

**Authors:** Yuki Miyamoto, Tomohiro Torii, Akito Tanoue, Junji Yamauchi

**Affiliations:** 1 Department of Pharmacology, National Research Institute for Child Health and Development, Setagaya, Tokyo 157-8535, Japan; 2 Laboratory of Molecular Neuroscience and Neurology, School of Life Sciences, Tokyo University of Pharmacy and Life Sciences, Hachioji, Tokyo 192-0392, Japan

## Abstract

Oligodendrocytes differentiate to wrap their plasma membranes around axons, forming the myelin sheath. A neuronal cue is one of the regulator elements controlling this process. Here, we demonstrate that VCAM1, which plays a key role throughout the immune system, is also expressed in oligodendrocytes, where it regulates the initiation of myelination. VCAM1 knockout mice exhibit reduced myelin thickness. Decreased myelin thickness is also observed in mutant mice of α4 integrin, which is a neuronal VCAM1 ligand. Furthermore, CD69 is identified as one of the transcripts downregulated when VCAM1 is knocked down in oligodendrocytes. Knockdown of CD69 in mice indicates its role in myelination. Therefore, VCAM1 contributes not only to the initiation of myelination but also to its regulation through controlling the abundance of CD69, demonstrating that an intercellular molecule whose primary role is in the immune system can also play an unexpected role in the CNS.

## Abstract (web summary)

The vascular cell adhesion molecule VCAM1 plays a role in the immune system but is also expressed in oligodendrocytes. Here, the authors find VCAM1 interacts with neuronal α4 integrin to regulate oligodendrocyte differentiation and thereby myelination, an effect mediated by downstream CD69 signalling.

## Graphical abstract

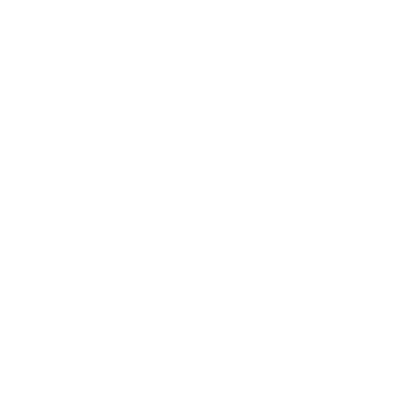

During development, and sometimes even during adulthood, oligodendrocyte precursor cells in the central nervous system (CNS) transition through multiple developmental stages^1^,^2^,^3^,^4^. The stages before birth involve cell division and migration along neuronal axons to their final destinations; after birth, these cells differentiate into oligodendrocytes. Like other glial cells, oligodendrocytes provide trophic and metabolic support to neurons^5^,^6^; they alone, however, wrap their plasma membranes around axons to form the myelin sheath. The mature myelin sheath with its multiple layers insulates axons to increase their nerve conduction velocity and protect them from various stresses including physical ones.

Myelin formation begins with the first contact of oligodendrocyte processes along axons and requires continuous communication between oligodendrocytes and axons^7^. Although we know of several intercellular interactive molecules that are important for the myelination process, the overall picture remains to be clarified. To date, the cell-adhesion molecules that control myelination have been discussed in terms of their effects on neurons rather than on oligodendrocytes. For example, in mouse experiments, negative signals have been well established^8^. The polysialylated form of neural cell-adhesion molecule (PSA-NCAM) 1 is specifically expressed in neurons, and PSA-NCAM1 negatively regulates myelination via oligodendrocyte NCAM1. In culture experiments, the immunoglobulin superfamily member L1 molecule is abundantly expressed in neurons and plays a role in initiating myelination by oligodendrocytes, possibly through their *trans* homophilic interaction^9^.

Our experiments using microarray analysis to investigate the cell-adhesion molecules in primary oligodendrocytes have revealed that vascular cell-adhesion molecule (VCAM)1 is one of their transcripts. VCAM1 is structurally composed of evolutionally conserved, immunoglobulin-like domains and is studied throughout the immune system^10^,^11^,^12^. It is now thought that VCAM1 is expressed in certain specific types of cells, such as endothelial cells, where it regulates cellular functions such as adhesion and morphological changes. The major VCAM1 ligand is known to be specific integrin components α4β1 (also called the very late antigen (VLA) 4). The receptor-ligand interaction is supported by *in vivo* evidence showing that complete deletion of VCAM1 results in a phenotype similar to that of α4 integrin; this phenotype is embryonically lethal due to a common developmental abnormality^13^,^14^,^15^. Herein, we report that the unit consisting of VCAM1 and α4 integrin also plays a role in oligodendrocyte-neuronal interaction. In fact, this unit positively regulates the initiation of myelination. Knockout of VCAM1 in oligodendrocytes leads to decreased myelin thickness in mice. A similar phenomenon is seen in α4 integrin mutant mice, consistent with the results of experiments using rat oligodendrocyte-neuronal co-cultures. Furthermore, we find that cluster of differentiation (CD)69 is simultaneously downregulated in association with knockdown of VCAM1 in oligodendrocytes, and that CD69, like VCAM1, is involved in the initiation of myelination. These results provide a new example of an intercellular molecular interaction that is involved in the regulation of both the immune system and the nervous system.

## Results

### Expression levels of VCAM1 and α4 integrin in the CNS

VCAM1 is an immunoglobulin-like type I transmembrane adhesion molecule that has been extensively investigated in the context of inflammatory and vascular biological events^10^,^11^,^12^. Our microarray analysis using rat primary oligodendrocytes revealed that VCAM1 is transcribed in these cells (Supplementary Table 1).

**Figure 1:**
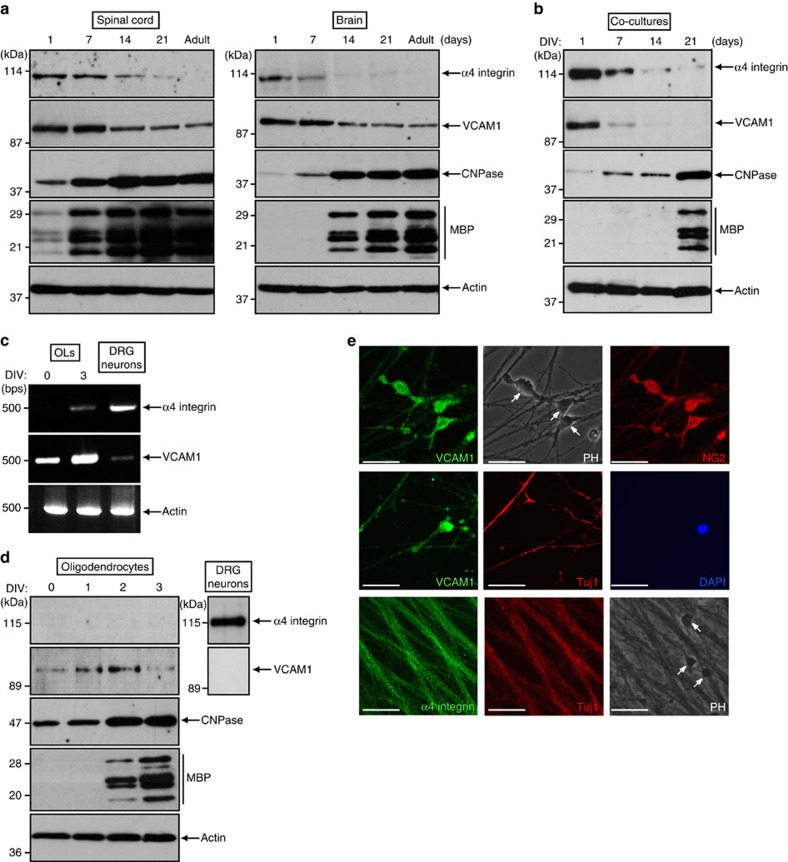
Changes in expression levels of VCAM1 and α4 integrin. (**a**) Tissue extracts were prepared from mouse spinal cords or whole brains on postnatal days 1–21 or in adulthood, subjected to SDS-polyacrylamide gel electrophoresis, transferred to a PVDF membrane and immunoblotted with an antibody against α4 integrin, VCAM1, CNPase, MBP or control actin. Data are representative of four experiments. (**b**) Rat oligodendrocytes and DRG neurons were co-cultured for 1–21 DIV, lysed and used for the respective immunoblottings. Data are representative of three experiments. (**c**) RT-PCR analysis was performed for α4 integrin, VCAM1 or control actin in differentiating oligodendrocytes (OLs) or neurons. Data are representative of two experiments. (**d**) Oligodendrocytes or neurons were lysed and used for immunoblottings. Data are representative of three experiments. (**e**) DIV2 co-cultures were co-stained with antibodies against VCAM1 (green) and NG2 (red; top row), VCAM1 (green), Tuj1 (red) and DAPI (middle row), or α4 integrin (green) and Tuj1 (red) (bottom row). PH, phase-contrast microscopy. Arrows indicate oligodendrocyte cell bodies. Data are representative of three experiments. Scale bar, 50 μm.

Immunoblotting analysis showed that, in the developing spinal cords and whole brains of mice, expression of VCAM1 was clearly detected until around 7 days after birth after which it gradually decreased (Fig. 1a). Similar changes in expression levels were observed in the α4 integrin subunit of the major VCAM1 ligand. The process of myelination in the spinal cord and brain begins within one week after birth and remains very active until around 1 month of age^1^,^2^, as evidenced by changes in the expression levels of the myelin marker proteins 2′, 3′-cyclicnucleotide 3′-phosphodiesterase (CNPase) and myelin basic protein (MBP). The changes in expression of VCAM1 and α4 integrin in co-cultures established from primary oligodendrocytes and primary dorsal root ganglion (DRG) neurons (1–21 days *in vitro* (DIV)) of rats were similar to those observed in developing spinal cords and brains (Fig. 1b). These results suggest the involvement of a possible interaction of VCAM1 with α4 integrin in myelination, especially in its initiation.

RT-PCR analysis using RNAs isolated from differentiating oligodendrocytes or neurons showed that VCAM1 was primarily transcribed in oligodendrocytes and that α4 integrin was primarily transcribed in neurons (Fig. 1c), consistent with the results of immunoblottings using the respective cell culture lysates (Fig. 1d). We also co-stained DIV2 co-cultures with an anti-VCAM1 or α4 integrin antibody, together with an oligodendrocyte lineage cell or neuronal marker antibody (NG2 or βIII-tubulin (also called Tuj1), respectively). In DIV2 co-cultures, expression of VCAM1 was detected primarily in NG2-positive cells (Fig. 1e, top row) and only weakly in Tuj1-positive neuronal fibres (Fig. 1e, middle row). In contrast, Tuj1-positive neurons were co-stained with α4 integrin whose expression was not detected in oligodendrocyte cell bodies (Fig. 1e, bottom row). These results suggest a relationship between VCAM1 on oligodendrocytes (as the receptor) and α4 integrin on neurons (as the ligand).

This possible relation can be supported by immunohistochemical data using spinal cord cross sections. Platelet-derived growth factor receptor (PDGFR)α-positive oligodendrocyte precursor cells and CNPase-positive pre-oligodendrocytes were co-stained with an anti-VCAM1 antibody at postnatal days 3 and 14, respectively. In CC1-positive mature myelinating oligodendrocytes at postnatal day 21, the percentages of cells expressing VCAM1 were decreased (Supplementary Fig. 1a,b). VCAM1 was neither co-stained with an anti-glial fibrillary acidic protein (GFAP; astrocyte marker), nor was it co-stained with an anti-PDGFRβ (pericyte marker) antibody (Supplementary Fig. 1c,d). On the other hand, α4 integrin-positive regions were often found in proximity to regions labelled with an anti-NeuN antibody and were surrounded by CC1-positive regions (Supplementary Fig. 1e).

### VCAM1 regulates proliferation and differentiation

**Figure 2:**
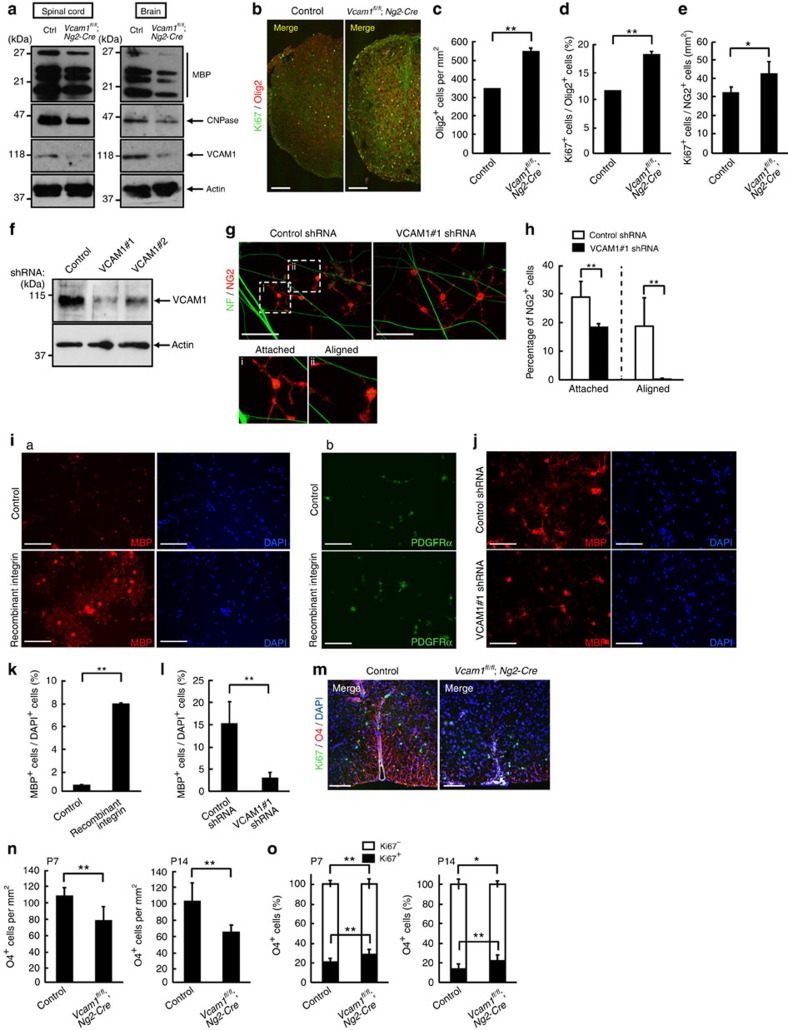
VCAM1 is involved in the regulation of proliferation and morphological changes in oligodendrocytes. (**a**) Tissue lysates from 7-day-old NG2-Cre-driven VCAM1 conditional knockout (*VCAM1*^*fl/fl*^*; Ng2-Cre*) or control (Ctrl) mouse spinal cords or 11-day-old whole brains were immunoblotted with an antibody against MBP, CNPase, VCAM1 or actin. Data are representative of three experiments. (**b**–**d**) Antibodies against Ki67 (green) and Olig2 (red) were used for co-staining in 2-day-old spinal cord cross sections. Data are representative. The scale bars indicate 200 μm. The number of Olig2^+^ cells per one square millimetre was counted. Data were evaluated using Student’s *t*-test (**P=0.000528; *n*=19 slices of two independent experiments). The percentage of Ki67^+^ cells among the Olig2^+^ cells is shown. Data were evaluated using Student’s *t*-test (**P=0.00807; *n*=6 slices of two independent experiments). (**e**) Antibodies against Ki67 and NG2 were used for co-staining in 2-day-old spinal cord. The percentage of Ki67^+^ cells among the NG2^+^ cells per one square millimetre is shown. Data were evaluated using Student’s *t*-test (*P=0.0275; *n*=6 slices of two independent experiments). (**f**–**h**) Knock-down efficiencies of shRNAs were confirmed by immunoblotting. Data are representative of three experiments. Oligodendrocytes were transfected with VCAM1 shRNA or control, co-cultured with neurons for 2 days, and co-stained with antibodies against NF (green) and NG2 (red). Magnified images of the dotted boxes (i, attached; ii, aligned) are shown below. Data are representative. Scale bar, 100 μm. The percentage of NG2^+^ cells whose process tips or cell bodies were attached to axons or aligned along axons is shown. Data were evaluated using Student’s *t*-test (**P=0.00109 (attached cells) or *P*=0.00982 (aligned cells); *n*=6 areas of three experiments). (**i**,**k**) Oligodendrocytes were cultured on dishes coated with or without recombinant α4β1 integrin in a growth medium containing PDGF and bFGF. Cells were co-stained with an anti-MBP antibody (red) and DAPI (blue) (a) or an anti-PDGFRα antibody (green) (b). Data are representative. Scale bar, 100 μm. The percentage of MBP^+^ cells is shown. Data were evaluated using Student’s *t*-test (**P=1.14E−10; *n*=6 areas of two independent experiments). (**j**,**l**) Oligodendrocytes transfected with VCAM1#1 or control shRNA were cultured on recombinant α4β1 integrin-coated dishes in the differentiation medium and co-stained with an anti-MBP antibody (red) and DAPI (blue). Data are representative. Scale bar, 100 μm. The percentage of MBP^+^ cells is shown. Data were evaluated using Student’s *t*-test (**P=1.82E−21; *n*=6 areas of two independent experiments). (**m**–**o**) Antibodies against Ki67 (green) and O4 (red) were used for co-staining in 7-day-old spinal cord. DAPI staining is also shown. Data are representative. Scale bar, 100 μm. The number of O4^+^ cells was counted. Data were evaluated using Student’s *t*-test (**P=5.87E−05 (P7) or *P*=5.87E−06 (P14); *n*=12 slices of two independent experiments). The percentage of Ki67^−^ or Ki67^+^ cells among the O4^+^ cells is shown. Data were evaluated using Student’s *t*-test (**P=0.00344 (P7, Ki67^−^), 0.00344 (P7, Ki67^+^) or 0.000580 (P14, Ki67^+^) *P=0.0494 (P14, Ki67^−^); *n*=12 slices of two independent experiments).

We investigated the effect of the loss of VCAM1 in oligodendrocytes. We generated VCAM1 conditional knockout (cKO) mice by crossing VCAM1 floxed mice with NG2 promoter-controlled Cre recombinase transgenic mice^16^ (see Supplementary Fig. 2a–c). In VCAM1^flox/flox^; NG2-Cre^+/−^ mice (tentatively referred to as *VCAM1*^*fl/fl*^*; Ng2-Cre* mice in figure panels), expression levels of VCAM1 as well as the other myelin marker proteins in spinal cords and brains were markedly decreased compared with littermate controls (VCAM1^flox/flox^; NG2-Cre^−/−^; Fig. 2a). To examine changes in oligodendrocyte proliferation in spinal cord sections using VCAM1 cKO mice and controls, we doubly labelled them with the cell proliferation antigen Ki67 and the oligodendrocyte lineage cell marker Olig2 on postnatal days 2, 7, 14 and 21 (Fig. 2b). On postnatal days 2, 7 and 14, knockout of VCAM1 in oligodendrocytes increased both the number of Olig2-positive (Olig2^+^) cells and the percentage of proliferating Olig2-positive (Ki67^+^/Olig2^+^) cells (Fig. 2c,d, and Supplementary Fig. 3a,b). The number of proliferating, NG2-positive (Ki67^+^/NG2^+^) cells in VCAM1 cKO mice was also increased (Fig. 2e), suggesting that an increase in Ki67^+^/Olig2^+^ cells can be due to an increase in oligodendrocyte precursor cells. In 21-day-old mice, Olig2-positive cell numbers and the percentage of proliferating cells were comparable in control and VCAM1 cKO mice (Supplementary Fig. 3a,b).

To further examine the effect of VCAM1 ablation on oligodendrocyte generation during development, we carried out 5-bromo-2′-deoxyuridide (BrdU) pulse-chase analysis. The percentage of BrdU-positive cells expressing CC1 (BrdU^+^/CC1^+^) cells in VCAM1 cKO mice was increased (Supplementary Fig. 4a,b). Collectively, these findings show that VCAM1 is likely to be involved in adjusting the number of oligodendrocyte lineage cells *in vivo*.

Next, we explored whether VCAM1 affects the initial contact of oligodendrocyte processes along axons. We evaluated the percentage of NG2-positive (NG2^+^) cells that were attached to neurofilament-positive (NF^+^) axons or aligned along them in DIV2 co-cultures. We made two non-overlapping VCAM1 short-hairpin RNAs (shRNAs) (VCAM1#1 and VCAM1#2). As the knockdown efficiency of VCAM1#1 was greater than that of VCAM1#2 (Fig. 2f), we used VCAM1#1 in the following experiments. We typically observed that 28.9±5.54% of NG2^+^ cells had their process tips or cell bodies attached to axons. In all, 18.7±9.98% of NG2^+^ cells were aligned along axons (Fig. 2g,h). In contrast, VCAM1 knockdown decreased these values by >25% and >90%, respectively. VCAM1 is needed for the initial contact of oligodendrocyte processes to axons *in vitro*.

Accordingly, we next examined whether VCAM1 has an effect on oligodendrocyte differentiation. We spread out primary oligodendrocytes on plastic dishes coated with or without recombinant α4β1 integrin, cultured these cells in an oligodendrocyte growth medium containing PDGF and FGF for 3 days, and immunostained them with an anti-MBP (Fig. 2i(a)) or PDGFRα (Fig. 2i(b)) antibody. Even under oligodendrocyte growth conditions the number of MBP-positive (MBP^+^) cells with myelin web-like structures was greater in α4β1 integrin-coated dishes (Fig. 2i,k; 7.95±0.0403% in α4β1 integrin-coated dishes, compared with 0.575±0.00917% in control dishes). The number of PDGFRα-positive cells was comparable (Fig. 2i(b)). On the other hand, VCAM1 knockdown decreased the number of MBP^+^ cells in α4β1 integrin-coated dishes by ∼80% (Fig. 2j,l), revealing that α4β1 integrin ligation of VCAM1 promotes oligodendrocyte differentiation *in vitro*.

Furthermore, to investigate the effect of VCAM1 ablation on oligodendrocyte differentiation *in vivo*, Ki67-positive and -negative cells labelled with the oligodendrocyte differentiation marker O4 were analysed at postnatal days 7 and 14. The percentage of double-positive cells (Ki67^+^/O4^+^) was increased in VCAM1 cKO mice (Fig. 2m,o). On the other hand, the percentage of non-proliferating O4-positive (O4^+^) cells (Ki67^−^/O4^+^) and the number of O4^+^ cells were both decreased in VCAM1 cKO mice (Fig. 2n,o). It is thus thought that VCAM1 knockout increases cell proliferation and thereby delays the oligodendrocyte differentiation process *in vivo*.

### VCAM1 and the ligand interaction promotes myelination

We established a long-term oligodendrocyte-neuronal co-culture system that would allow us to assay myelination. Knockdown of VCAM1 in oligodendrocytes inhibited formation of MBP-positive myelin segments (Supplementary Fig. 5a,b). In keeping with this finding, MBP expression was decreased by VCAM1 knockdown (Supplementary Fig. 5c), suggesting that VCAM1 plays a role in initiating myelination *in vitro*.

We also checked the effect of a VCAM1 extracellular domain’s fusion protein (VCAM1-Fc) and that of an anti-α4 integrin antibody, each of which was expected to block the VCAM1 ligand, on co-cultures. Each of these proteins inhibited myelination by oligodendrocytes (Supplementary Fig. 5d–f). A similar effect was observed when an anti-VCAM1 antibody was added to co-cultures, indicating the importance of the interaction between oligodendrocyte VCAM1 and its ligand in myelination. Under these conditions, the amounts of neurofilaments were comparable among the respective experiments.

We transfected oligodendrocytes with the plasmid encoding VCAM1 or with vector only then plated them on DRG neurons, which express α4 integrin, in an oligodendrocyte growth medium. Transfection of VCAM1 increased the percentage of Olig2^+^ cells with MBP-positive myelin segments (Supplementary Fig. 6a,b). Considering these findings together, we conclude that VCAM1 itself has the ability to promote differentiation and thereby to trigger myelination *in vitro*.

### VCAM1 is required for the initiation of myelination

**Figure 3:**
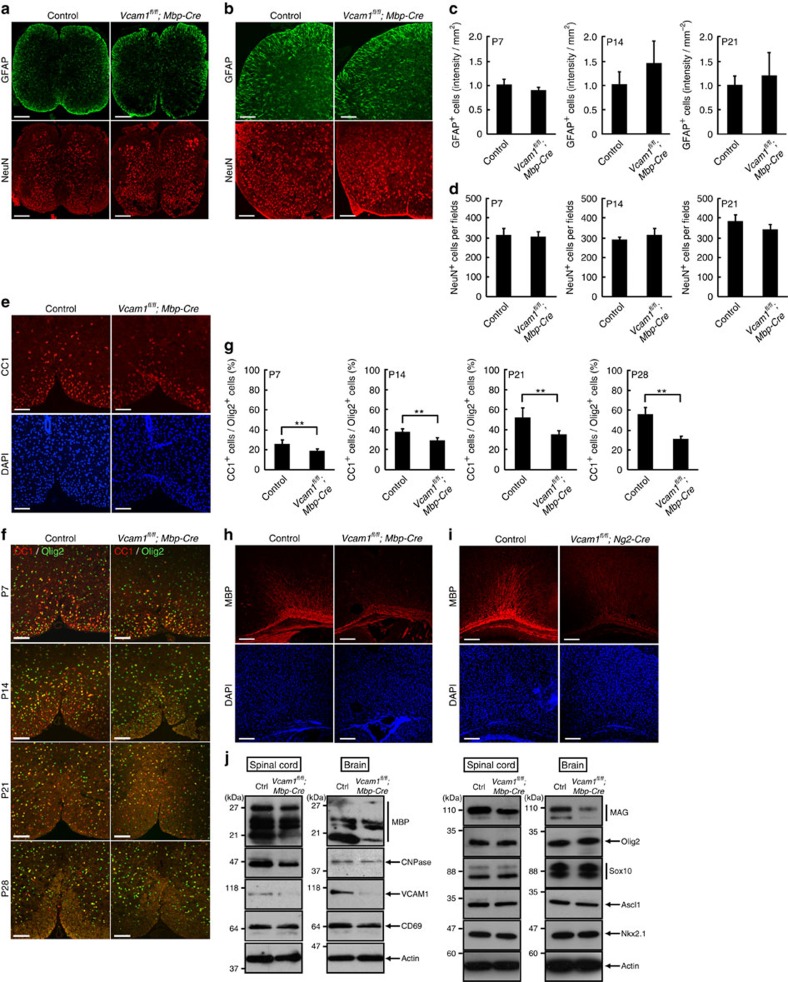
Knockout of VCAM1 in oligodendrocytes decreases myelin marker protein expression in mice. (**a**,**b**) MBP-Cre-driven VCAM1 conditional knockout (*VCAM1*^*fl/fl*^*; Mbp-Cre*) and control mouse spinal cord cross sections at postnatal day 7 were stained with an antibody against GFAP (green) or NeuN (red). Data are representative. Scale bar, 200 μm (**a**); 100 μm (**b**). (**c**) The intensity of GFAP staining per one square millimetre was semi-quantified at postnatal days 7, 14 and 21. Data were evaluated using Student’s *t*-test (non-significance; *n*=6 slices of two independent experiments). (**d**) The number of NeuN^+^ cells per field was counted. Data were evaluated using Student’s *t*-test (non-significance; *n*=5 slices of two independent experiments). (**e**) Spinal cord cross sections of *VCAM1*^*fl/fl*^*; Mbp-Cre* and control mice at postnatal day 7 were co-stained with an antibody against CC1 (red) and DAPI (blue). Data are representative. Scale bar, 100 μm. (**f**,**g**) Antibodies against CC1 (red) and Olig2 (green) were used for co-staining in spinal cord cross sections at postnatal days 7, 14, 21 and 28. Data are representative. Scale bar, 100 μm. The percentage of CC1^+^ cells among the Olig2^+^ cells is shown. Data were evaluated using Student’s *t*-test (**P=0.00638 (P7), 0.000961 (P14), 0.00111 (P21) or 2.11E-09 (P28); *n*=6–10 slices of two independent experiments). (**h**,**i**) Corpus callosum sections of *VCAM1*^*fl/fl*^*; Mbp-Cre* and control mouse (**h**) or *VCAM1*^*fl/fl*^*; Ng2-Cre* and control mouse (**i**) at postnatal day 11 were co-stained with an anti-MBP antibody (red) and DAPI (blue). Data are representative of six slices of two independent experiments. Scale bar, 200 μm. (**j**) Tissue lysates from 7-day-old spinal cords or 11-day-old brains were immunoblotted with an antibody against MBP, CNPase, VCAM1, CD69, MAG, Olig2, Sox10, Ascl1, Nkx2.1 or actin. Data are representative of three experiments.

To assess the *in vivo* role of VCAM1 in oligodendrocyte myelination, we crossed VCAM1 floxed mice with MBP promoter-controlled Cre transgenic mice^17^. We first performed immunohistochemical analyses of the astrocyte marker GFAP and the neuron marker NeuN in 7-, 14- and 21-day-old spinal cords. We failed to observe obvious differences between VCAM1^flox/flox^; MBP-Cre^+/−^ mice (tentatively referred to as *VCAM1*^*fl/fl*^*; Mbp-Cre* mice in figure panels) and controls (Fig. 3a–d). CC1 staining, on the other hand, was decreased in the spinal cords of 7-day-old VCAM1 cKO mice (Fig. 3e). The percentage of CC1 and Olig2 double-positive cells (CC1^+^/Olig2^+^) in VCAM1 cKO mice decreased throughout the first 28 postnatal days (Fig. 3f,g). Similarly, knockout of VCAM1 decreased MBP staining levels in 11-day-old corpus callosum (Fig. 3h). NG2 promoter-controlled Cre-driven VCAM1 cKO mice also exhibited decreased MBP expression (Fig. 3i). To further confirm the changes in myelin marker expression levels through immunoblotting, we isolated total proteins from spinal cords or brains. Knockout decreased the expression levels of MBP, CNPase and MAG (Fig. 3j), suggesting that VCAM1 plays a role in myelination *in vivo*. The expression levels of Olig2, Sox10, Ascl1 and Nkx2.1, the master transcription factors controlling oligodendrocyte lineage cell fate, were comparable in VCAM1 cKO and control mice.

We also crossbred VCAM1 floxed mice with tamoxifen (TAM)-inducible PLP1 promoter-controlled Cre transgenic mice (tentatively referred to as *VCAM*^*fl/fl*^*; Plp1-Cre*^*ERT*^ mice in figure panels). The expression level of VCAM1 was decreased in brain lysates (Supplementary Fig. 7a). Inducible knockout of VCAM1 decreased the percentage of CC1 and Olig2 double-positive cells (CC1^+^/Olig2^+^) in the spinal cords and the MBP staining levels in the corpus callosum (Supplementary Fig. 7b–e). Similarly, inducible knockout of VCAM1 decreased the expression levels of MBP and CNPase (Supplementary Fig. 7a). These findings suggest that VCAM1 can also be involved in the regulation of myelination in adulthood.

**Figure 4:**
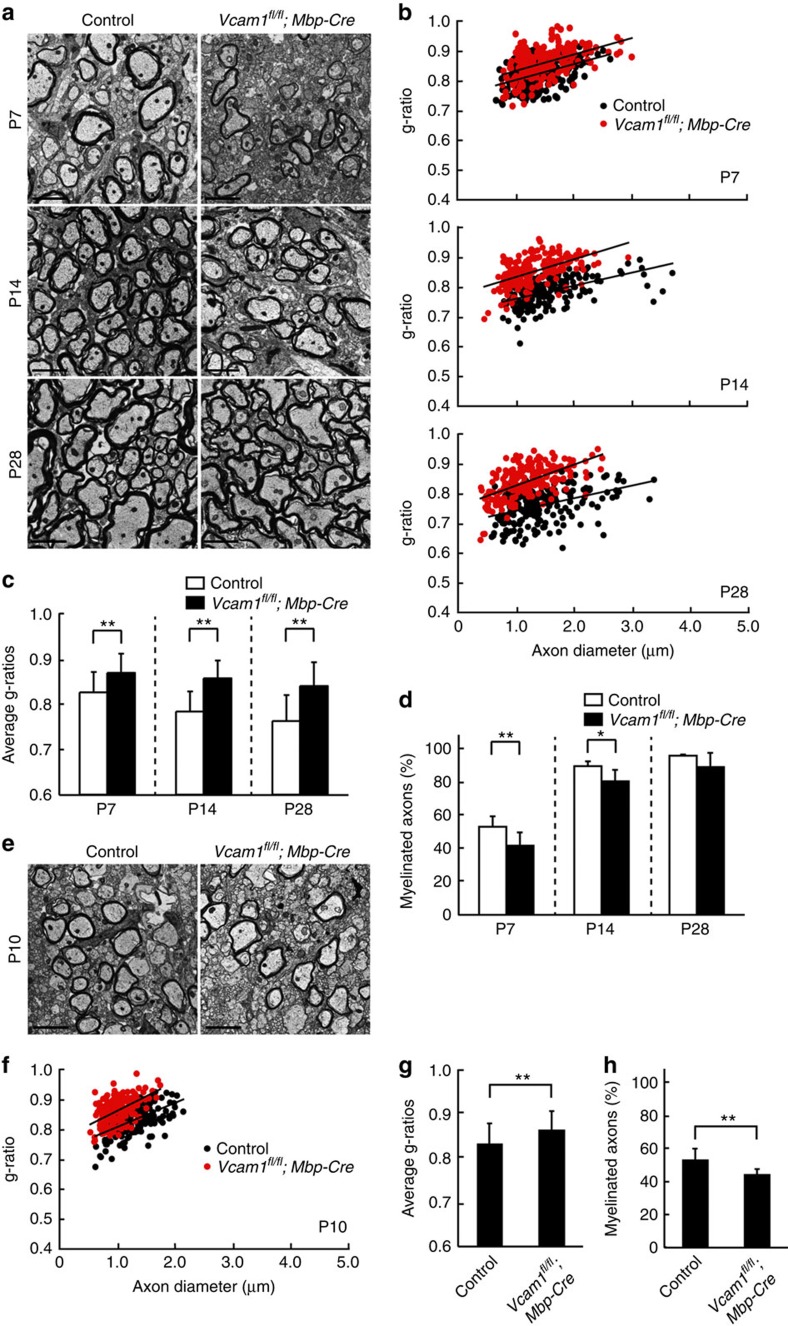
Knockout of VCAM1 results in decreased myelin thickness during postnatal development. (**a**–**d**) Electron microscopic data of 7-, 14- and 28-day-old (P7, P14 and P28) spinal cords are shown. Data are representative. Scale bar, 2 μm. The g-ratios (200–300 myelinated fibres) are plotted for axon diameters. Average g-ratios are also shown. Data were evaluated using Student’s *t*-test (**P=1.54E−21 (P7), 1.39E−48 (P14) or 3.50E−37 (P28); *n*=208–282 axons of two independent experiments). The percentage of myelinated axons is indicated. Data were evaluated using Student’s *t*-test (**P=0.00963 (P7), *P=0.0135 (P14) or non-significance (P28); *n*=6–8 areas of two independent experiments). (**e**–**h**) Electron microscopic data of 10-day-old corpus callosum are shown. Data are representative. Scale bar, 2 μm. The g-ratios (200–300 myelinated fibres) are plotted for axon diameters. Average g-ratios are also shown. Data were evaluated using Student’s *t*-test (**P=3.47E−10; *n*=209–224 axons of two independent experiments). The percentage of myelinated axons is indicated. Data were evaluated using Student’s *t*-test (**P=0.00523; *n*=8 areas of two independent experiments).

Electron microscopic analysis revealed that VCAM1 cKO mice exhibited decreased myelin thickness in the spinal cord compared with controls (Fig. 4a). These findings are clearly evident from quantification of the average g-ratios, which are the numerical ratios between the axon diameters and the outer diameters of the myelinated fibres. Larger ratios indicate thinner myelin sheaths. At an early postnatal stage (day 7), the average g-ratios were 0.868±0.0434 in VCAM1 cKO mice and 0.825±0.0462 in controls (Fig. 4b, upper panel; Fig. 4c, left bars). At postnatal day 14, the average g-ratios were 0.857±0.0397 in VCAM1 cKO mice and 0.783±0.0452 in controls (Fig. 4b, middle panel; Fig. 4c, middle bars). Finally, at postnatal day 28, the average g-ratios were 0.839±0.0534 in knockout mice and 0.762±0.0577 in controls (Fig. 4b, bottom panel; Fig. 4c, right bars), revealing an overall trend in which myelin sheaths gradually thickened in controls but barely changed in VCAM1 cKO mice. In addition, the percentage of myelinated axons was decreased in VCAM1 cKO mice than in controls at postnatal days 7 and 14 (Fig. 4d). The values observed in our controls are in agreement with those reported previously^18^. With regard to the idea that VCAM1 promotes myelination, results similar to those of ultrastructural studies have been observed in oligodendrocyte-neuronal co-culture studies. In the corpus callosum, knockout mice consistently exhibit decreased myelin thickness compared with controls (Fig. 4e). The average g-ratios at postnatal day 10 were 0.864±0.0429 in VCAM1 cKO mice compared with 0.831±0.0477 in controls (Fig. 4f,g). The percentage of myelinated axons was also decreased in VCAM1 cKO mice (Fig. 4h). Aside from this difference in myelin thickness, the ultrastructures of the myelin sheath in the spinal cord and corpus callosum were apparently comparable in VCAM1 cKO and controls.

### α4 integrin is required for the initiation of myelination

Since α4 integrin knockout mice are embryonically lethal^15^, we used knockin mice with a partially defective biological function of α4 integrin in this study^19^. These mice possess the Arg-1007-to-Ala mutation in the conserved amino acid sequence (GFFKR^1007^) within the α4 integrin intracellular domain, where a salt bridge is formed with an integrin β subunit and is thought to be critical for controlling integrins’ activity^20^. The mutant mice are known to show blunted biological activity of α4 integrin^19^,^20^.

Using homozygous mice (Supplementary Fig. 8a,b) of the mutant α4 integrin allele (tentatively referred to as α4INTG KI in figure panels), we first characterized the Olig2-positive oligodendrocyte lineage cells in these mice. Both the number of Olig2-positive cells and the percentage of proliferating Olig2-positive (Ki67^+^/Olig2^+^) cells were increased (Supplementary Fig. 9a–c), consistent with the results from VCAM1’s genetically modified mice.

**Figure 5:**
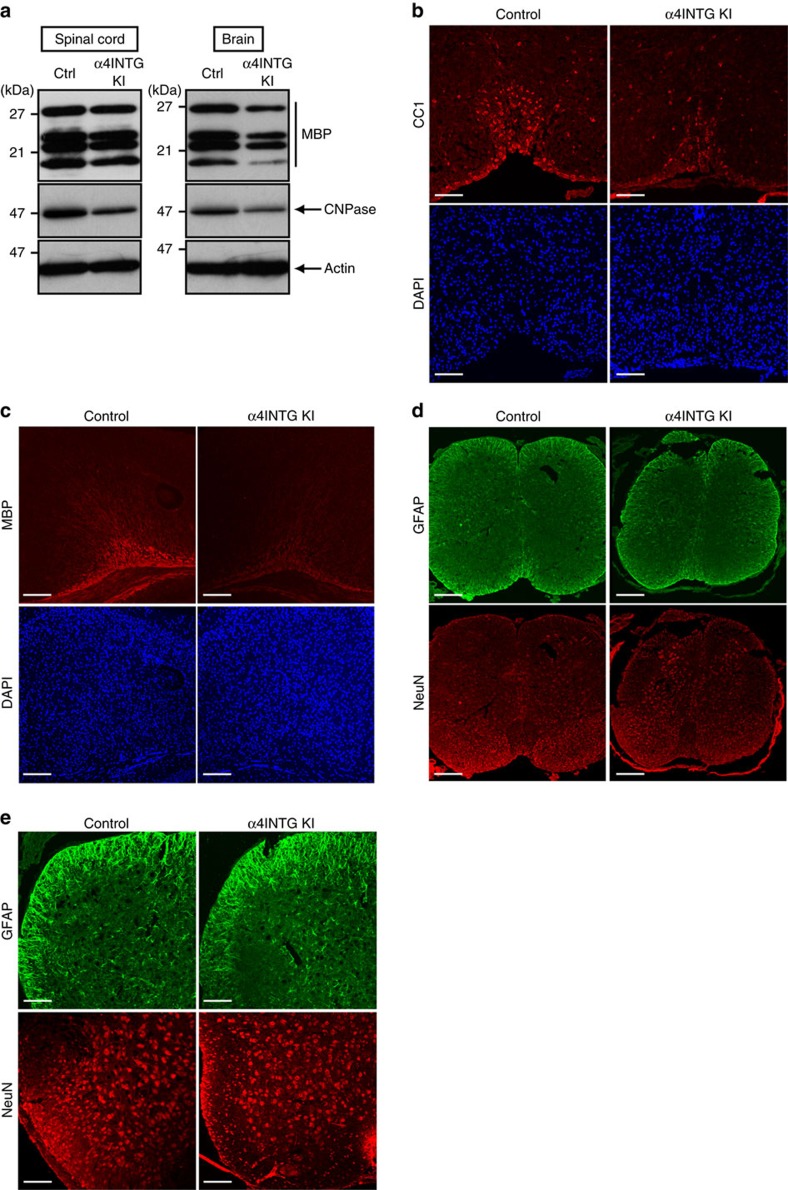
Mutant mice of the VCAM1 ligand subunit α4 integrin exhibit decreased myelin marker protein expression. (**a**) Tissue lysates from 7-day-old α4 integrin knockin mouse (α4INTG KI) spinal cords or 11-day-old brains were immunoblotted with an antibody against MBP, CNPase or actin. Data are representative of two experiments. (**b**) In spinal cord cross sections at postnatal day 7, CC1 (red) and DAPI (blue) staining data are shown. Data are representative of two experiments. Scale bar, 100 μm. (**c**) Corpus callosum sections at postnatal day 11 were co-stained with an anti-MBP antibody (red) and DAPI (blue). Data are representative of two experiments. Scale bar, 200 μm. (**d**,**e**) Spinal cord cross sections at postnatal day 7 were stained with an antibody against GFAP (green) or NeuN (red). Data are representative of two experiments. Scale bar, 200 μm (**d**) or 100 μm (**e**).

We assessed the expression levels of MBP and CNPase. The mutant mice exhibited decreased expression of both proteins in both spinal cords and brain tissues (Fig. 5a). These results are consistent with the decreased protein expression of CC1 and MBP in mutant mice as revealed through immunohistochemistry (Fig. 5b,c), suggesting that α4 integrin is involved in myelination. In contrast, the staining behaviours of the GFAP and NeuN antigens were comparable in mutant and control mice (Fig. 5d,e).

**Figure 6:**
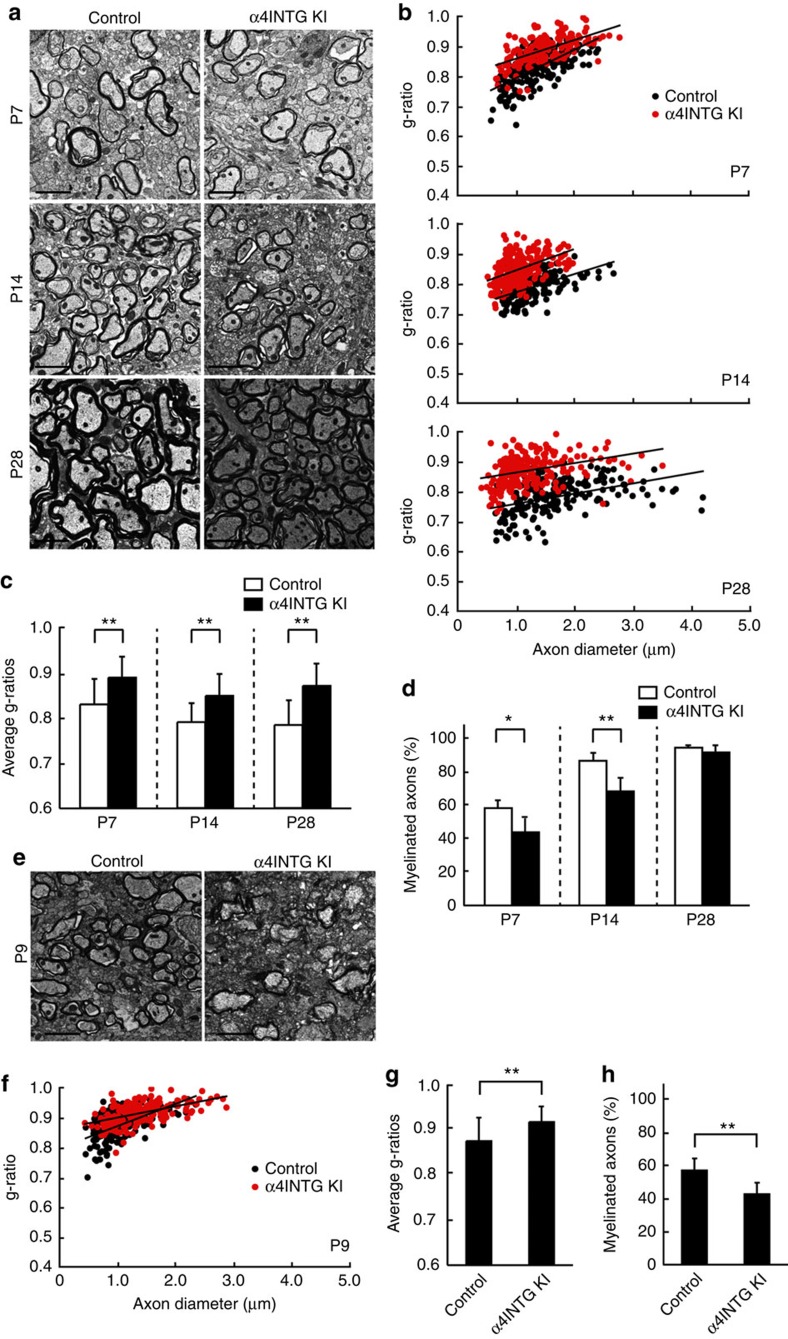
Mutant mice of α4 integrin exhibit decreased myelin thickness during postnatal development. (**a**–**d**) Electron microscopic data of 7-, 14- or 28-day-old spinal cords are shown. Data are representative. Scale bar, 2 μm. The g-ratios (200–300 myelinated fibres) are plotted for axon diameters. Average g-ratios are also shown. Data were evaluated using Student’s *t*-test (**P=5.59E−25 (P7), 4.22E−31 (P14) or 3.54E−50 (P28): *n*=212–239 axons of two independent experiments). The percentage of myelinated axons is indicated. Data were evaluated using Student’s *t*-test (**P=0.000420 (P14), *P=0.0105 (P7) or non-significance (P28); *n*=6–8 areas of two independent experiments). (**e**–**h**) Electron microscopic data of 9-day-old corpus callosum are shown. Data are representative. Scale bar, 2 μm. The g-ratios (200–300 myelinated fibres) are plotted for axon diameters. Average g-ratios are also shown. Data were evaluated using Student’s *t*-test (**P=1.50E−16; *n*=196–202 axons of two independent experiments). The percentage of myelinated axons is indicated. Data were evaluated using Student’s *t*-test (**P=0.00305; *n*=7–8 areas of two independent experiments).

We next analysed the myelin sheath ultrastructure by electron microscopy, and observed that mutant mice exhibited decreased myelin thickness in the spinal cord compared with controls (Fig. 6a). These findings are clearly evident from quantification of the average g-ratios. At postnatal day 7, the average g-ratios were 0.888±0.0457 in mutant mice and 0.828±0.0569 in controls (Fig. 6b, upper panel; Fig. 6c, left bars). At postnatal days 14 and 28, the average g-ratios were 0.848±0.0484 and 0.870±0.0493, respectively, in mutant mice, and 0.789±0.0434 and 0.783±0.0554, respectively, in controls (Fig. 6b, middle and bottom panels; Fig. 6c, middle and right bars). The percentage of myelinated axons was decreased in mutant mice, especially in the early stages (Fig. 6d). These values correlate with the results from VCAM1 cKO mice. Mutant mice exhibited decreased myelin thickness compared with controls in the corpus callosum as well (Fig. 6e). In this region of the brain, the average g-ratios at postnatal day 9 were 0.917±0.0352 in mutant mice compared with 0.875±0.0491 in controls (Fig. 6f,g). The percentage of myelinated axons in mutant mice was also decreased (Fig. 6h). In the spinal cord and corpus callosum, myelin ultrastructures in mutant mice were apparently comparable to those in controls.

### CD69 is also involved in the initiation of myelination

We have identified CD69 as one of the gene products downregulated in oligodendrocytes with knocked-down VCAM1 (Supplementary Table 2). CD69 is a member of the C-type lectin disulfide-linked membrane protein family^21^. It is well-known that CD69 is a lymphocyte early activation marker^22^. Indeed, knockout of VCAM1 downregulated the expression levels of CD69 proteins significantly though not completely (Fig. 3j). In controls, CD69 was ∼60% co-stained with CC1-positive cells, a degree of overlap similar to that of VCAM1; in contrast, VCAM1 knockout decreased CC1-positive cells (Supplementary Fig. 10a,b). On the basis of that finding, we set out to clarify whether CD69 is involved in oligodendrocyte myelination. We transfected an shRNA for CD69 into oligodendrocytes and established oligodendrocyte-neuronal co-cultures. Knockdown of CD69 inhibited formation of MBP-positive myelin segments by >80% (Supplementary Fig. 11a,b), consistent with the results obtained through immunoblotting with an anti-MBP antibody (Supplementary Fig. 11c). Also, expression levels of CD69 in brain were maintained into adulthood but were somewhat decreased (Supplementary Fig. 11d). Immunohistochemical data showed that PDGFRα- and CNPase-positive cells were co-stained with an anti-CD69 antibody, while in CC1-positive mature oligodendrocytes, the percentages of cells expressing CD69 were decreased (Supplementary Fig. 11e). These results suggested that CD69, like VCAM1, may act during the phase when the process of myelination occurs. In RT-PCR, CD69 was detected in oligodendrocytes but not in neurons, and was downregulated following induction of differentiation (Supplementary Fig. 11f).

Thus we transfected the plasmid encoding CD69 into oligodendrocytes under the VCAM1 knockdown background and co-cultured these cells with neurons. Transfection of CD69 reversed VCAM1 shRNA-mediated inhibition of myelin formation (Supplementary Fig. 12a,b). Furthermore, to confirm the rescue phenotype *in vivo*, we produced CD69 transgenic (Tg) mice. We injected a linearized transgene that contained the CD69 sequence under the control of an MBP promoter^23^ into fertilized mouse eggs according to the standard production method of Tg mice (Supplementary Fig. 13a–c). The Tg mice were crossbred with VCAM1 knockout mice. The Tg mice under the VCAM1 knockout background exhibited rescued CC1-staining knockout phenotypes (Supplementary Fig. 14a,b), consistent with the results from the *in vitro* rescue experiment. These results suggest that the effect of VCAM1 on oligodendrocyte myelination requires the presence of CD69.

**Figure 7:**
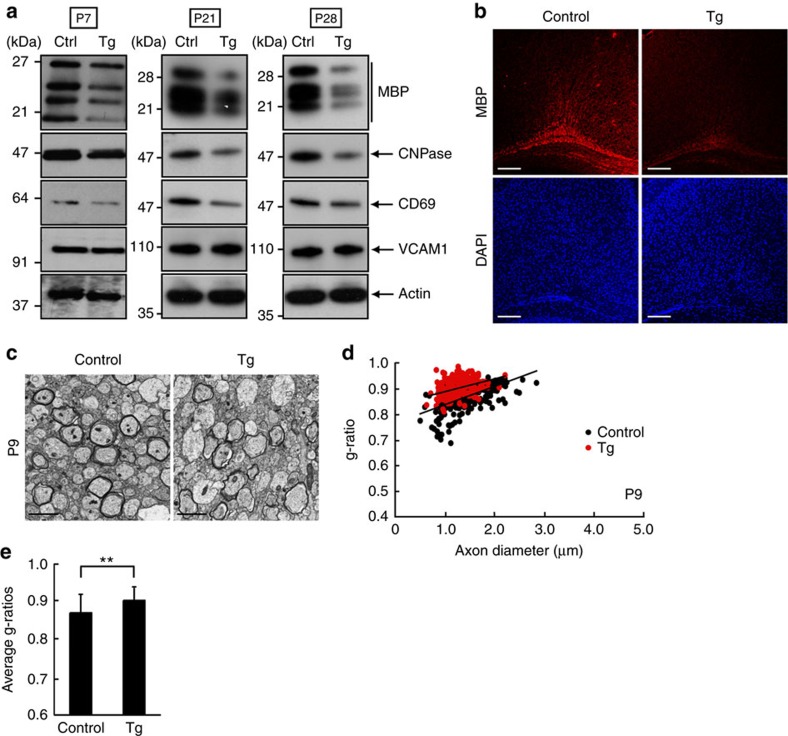
CD69 is also involved in the initiation of myelination. (**a**) Tissue lysates from 7-, 21- and 28-day-old CD69 shRNA transgenic (Tg) and control mouse brains were immunoblotted with an antibody against MBP, CNPase, CD69, VCAM1 or actin. Data are representative of three experiments. (**b**) Corpus callosum sections at postnatal day 11 were co-stained with an anti-MBP antibody (red) and DAPI (blue). Data are representative of two experiments. Scale bar, 200 μm. (**c**–**e**) Electron microscopic data of 9-day-old corpus callosum are shown. Data are representative. Scale bar, 2 μm. The g-ratios (200–300 myelinated fibres) are plotted for axon diameters. Average g-ratios are also shown. Data were evaluated using Student’s *t*-test (**P=7.13E−15; *n*=213–260 axons of two independent experiments).

We further analysed the role of CD69 *in vivo*. We injected fertilized mouse eggs with a linearized transgene that contained the CD69 shRNA inserted into an artificial miRNA backbone (Supplementary Fig. 15a–c). This knockdown construct is often called shRNAmir^24^,^25^. We succeeded in generating a Tg line in which CD69 was not very strongly knocked down, but the Tg mice of this line showed decreased expression levels of myelin marker proteins in brain tissues compared with nontransgenic littermate controls (Fig. 7a). These results were consistent with those from experiments in which MBP expression is immunohistochemically decreased (Fig. 7b). In contrast, CD69 knockdown did not obviously change expression levels of VCAM1 (Fig. 7a). In electron microscopic analysis, the transgenic mice exhibited decreased myelin thickness in the corpus callosum at postnatal day 10 (Fig. 7c–e; average g-ratio in knockout mice 0.916±0.0454 compared with 0.871±0.0491 in controls). With regard to the idea that CD69 promotes myelination, these results are consistent with the *in vitro* results concerning oligodendrocyte CD69.

Finally, to investigate possible mediators involved in the interaction of VCAM1 and CD69 in oligodendrocytes, we explored potential transcription factors. Since the positive regulators such as Olig2 and Sox10 were comparable in VCAM1 cKO and control mice (Fig. 3j), we checked whether the expression levels of Sox5 and/or Sox6, which have an opposite effect from that of Sox10 (ref. 26), were different in VCAM1 cKO mice. In VCAM1 cKO mice, the expression levels of Sox6 were increased whereas those of Sox5 appeared to be unchanged (Supplementary Fig. 16a). To examine the role of Sox6 in CD69 expression, we transfected Sox6 shRNA into primary oligodendrocytes and co-cultured the cells with primary neurons. Knockdown of Sox6 upregulated CD69 as well as MBP (Supplementary Fig. 16b). It is likely that VCAM1 maintains the abundance of CD69 by sustaining low levels of Sox6, which negatively regulates myelination by oligodendrocytes.

## Discussion

Myelination requires continuous and dynamic morphological changes of oligodendrocytes and their interaction with axons. Here, we show that VCAM1, a cell-adhesion molecule with multiple immunoglobulin-like domains, acts as the positive regulator of oligodendrocyte differentiation and the initiation of myelination (Supplementary Fig. 17). VCAM1 is well-studied in the immune system; in the CNS, it is primarily expressed in oligodendrocytes, while its specific ligand subunit, α4 integrin, is primarily expressed in neurons. Indeed, α4β1 integrin ligation of VCAM1 promotes differentiation in oligodendrocytes. In mice, knockout of VCAM1 in oligodendrocytes causes decreased myelin thickness compared with littermate controls. Similar results are observed in α4 integrin mutant mice, in keeping with the results from experiments with oligodendrocyte-neuronal co-cultures using an *in vitro* knockdown technique, a blocking antibody or a recombinant protein. When VCAM1 is knocked down in oligodendrocytes, CD69 is downregulated at the same time, possibly through Sox6. CD69 is also involved in the initiation of myelination, since knockdown decreases myelination *in vivo* as well as *in vitro*. It is thus suggested that, *in vivo*, the VCAM1 signal participates not only in initiating myelination through ligand ligation but also in mediating it by controlling the abundance of CD69. To date, little information is available concerning CD69’s intercellular and intracellular molecular network in the CNS; further studies on its role will enable us to understand the detailed mechanism by which VCAM1, acting with CD69, regulates the process of oligodendrocyte myelination.

In the immune system, VCAM1 is primarily expressed in endothelial cells while α4β1 integrin is known to be expressed in leucocytes. Their interaction in this context is well-studied with regard to a phenomenon called extravasation^27^. Under pathophysiological conditions, leucocytes actively migrate from the bloodstream into the tissues by crossing the endothelial monolayer lining along the vessels. Extravasation consists of three steps, namely, endothelial cell interaction with leucocytes, endothelial cell capture of leucocytes, and leucocyte migration into the underlying tissues through endothelial cell morphological changes^27^. Indeed, knockout of VCAM1 in mouse endothelial cells leads to reduced migration in α4β1 integrin-expressing immune cells including leucocytes^28^, as seen in mice lacking α4 integrin^29^. Similarly, inhibition of integrin function by a blocking antibody *in vitro* also suppresses their migration^30^. Endothelial VCAM1 in particular is likely to be involved in the early steps of extravasation, such as contact with other cells, adhering and capturing, all of which require continuous, situation-dependent morphological changes. Interestingly, it appears that cell morphological changes in the initial phases of myelination share some characteristics in common with those in the early phases of extravasation. Oligodendrocytes, which differentiate from oligodendrocyte precursor cells, grow multiple processes that attach to axons and eventually wrap them with myelin sheaths. It is thought that these processes require continuous, situation-dependent morphological changes. Thus the VCAM1 system can be counted among the examples in which similar molecular mechanisms are involved in the nervous system and the immune system. There is one striking difference, however, in the CNS, we have not detected an obvious difference in neuronal phenotypes between α4 integrin mutant mice and controls. In the CNS, therefore, unlike the immune system, the role of retrograde signalling from VCAM1 to integrin may vary depending on the interactive cell type.

It is known that the intracellular surface of VCAM1 specifically binds to the ezrin, radixin and moesin family proteins ezrin and moesin, localizing to microscopic membrane protrusions in endothelial cells^31^. These proteins generally connect receptors, including cell-adhesion molecules, to the actin cytoskeleton and this connection is crucial for cell morphogenesis. It is thought that ezrin and moesin act downstream of VCAM1 to form a cell–cell docking structure, in a process reminiscent of the attachment of oligodendrocytes to axons and their eventual wrapping of the axons with myelin sheaths. It is possible that, in oligodendrocytes, ezrin, radixin and moesin family proteins bind to VCAM1, forming an oligodendrocyte-neuronal docking structure.

Myelin-associated glycoprotein (MAG) is a well-known cell-adhesion molecule expressed in oligodendrocytes. It functions in the interactions between oligodendrocytes and axons. Deletion of MAG in mice causes not only an abnormal interaction between oligodendrocytes and axons, but also ultrastructural abnormalities of the myelin sheaths^32^,^33^,^34^. MAG is a member of the lectin-like molecules, which bind to sialylated glycoconjugates such as proteins and lipids; it functions both as a receptor for an axonal signal that helps oligodendrocytes to differentiate and myelinate, and as a ligand for a sialic acid-containing axonal receptor that is needed for the maintenance of myelinated axons in adulthood^35^. It is clear that CD69, another lectin family member, is involved in the initiation of oligodendrocyte myelination, but it will be interesting to explore whether CD69 has bidirectional properties similar to those of MAG.

In addition to the L1 molecule interaction in culture^9^ and the VCAM1 system demonstrated in the present study, it is known that Necl-1 (also called synaptic cell-adhesion molecule 3 (ref. 36) positively regulates myelination *in vivo*^37^. Yet the expression of Necl-1 is not observed in oligodendrocytes. Instead, Necl-1 is specifically expressed in neurons in the CNS. Since Necl-1 has properties indicating heterophilic as well as homophilic interactions, regulation of myelination by Necl-1 is likely to be mediated through other Necls that are expressed in oligodendrocytes^36^,^37^. Necl-4, for example, is upregulated following the myelination process and may be a potential partner for Necl-1 (ref. 37). It is likely that myelination utilizes more cell-adhesion molecules than we anticipated, perhaps because it involves continuous and dynamic cell–cell interactions. Many kinds of cell-adhesion molecules may be needed to initiate myelination and ensure that it proceeds in a spatio-temporal manner throughout development.

Evidence suggests that the VCAM1 system is involved in oligodendrocyte-neuronal interaction. Multiple sclerosis (MS) is a chronic inflammatory disease involving repeated demyelination and remyelination. In one of the earliest stages of MS, reactive T cells enter into CNS tissues^38^,^39^. Natalizumab is a humanized, therapeutic monoclonal antibody that specifically binds to α4 integrin, which is also expressed on inflammatory-activated T lymphocytes^40^. The antibody blocks the binding of activated T lymphocytes to brain endothelial VCAM1. Natalizumab thus reduces the severity of MS and related disorders. On the other hand, it has been suggested that patients receiving natalizumab treatment may be at risk for progressive multifocal leukoencephalopathy^41^. In this study, we propose the role of the VCAM1 system in oligodendrocyte-neuronal interaction. Although it is unknown whether the VCAM1 system is involved in remyelination under these pathophysiological conditions, it might be necessary to consider the potential risk to the oligodendrocytes that is posed by natalizumab. Further studies will promote our understanding of the precise mechanism by which signalling through VCAM1 regulates oligodendrocyte myelination. Such studies may also help us to elucidate a paradigm for remyelination and nerve regeneration.

## Methods

### Antibodies

The following antibodies were purchased: rabbit (sc-8304, 1:250) or goat polyclonal (sc-1504, 1:100) anti-VCAM1, rabbit polyclonal anti-α4 integrin (sc-14008, 1:250) and rabbit polyclonal anti-PDGFRα (sc-338, 1:25) from Santa Cruz Biotechnology (Santa Cruz, CA, USA); mouse monoclonal anti-βIII-tubulin (also called Tuj1, clone Tuj1, 1:300) and goat polyclonal anti-PDGFRβ (AF1042, 1:20) from R&D Systems (Minneapolis, MN, USA); rat monoclonal anti-VCAM1 (clone M/K-2, 1 μg ml^−1^), rat monoclonal anti-α4 integrin (clone PS/2, 1 μg ml^−1^), mouse monoclonal (clone 132.39, 1:500) or rabbit polyclonal (AB5320V, 1:1,000) anti-NG2, mouse monoclonal anti-MBP (clone SKB3, 1:500), mouse monoclonal anti-adenomatus polyposis coli (also called CC1, clone CC1, 1:1,000), mouse monoclonal anti-O4 (clone 81, 1:200), mouse monoclonal anti-NeuN (clone A60, 1:500) and mouse monoclonal anti-MAG (clone 513, 1:5,000) from Merck-Millipore (Billerica, MA, USA); mouse monoclonal anti-Ki67 antigen (9,449, 1:500) from Cell Signaling Technology (Danvers, MA, USA); rabbit polyclonal anti-α4 integrin (ab65984, 1:100), rabbit polyclonal anti-Olig2 (clone EPR26731, 1:500), rabbit polyclonal anti-Ki67 (clone SP-61, 1:500), rabbit polyclonal anti-Sox10 (clone EPR4007, 1:5,000), rabbit polyclonal anti-Ascl1 (ab38556, 1:500), rabbit polyclonal anti-Nkx2.1 (clone EPR5955(2), 1:5,000), rat monoclonal anti-BrdU (clone BU1/75 (ICR1), 1:40) and rabbit polyclonal anti-CD69 (ab136138, 1:100) from Abcam (Cambridge, UK); mouse monoclonal anti-MBP (SMI94, 1:100 or 1:1,000) and rabbit polyclonal anti-GFAP (PRB-571C, 1:3,000) from Covance (Princeton, NJ, USA); mouse monoclonal anti-CNPase (clone 11-501, 1:500 or 1:5,000) and rabbit polyclonal anti-neurofilament’s large subunit (N4142, 1:300) from Sigma-Aldrich (St Louis, MO, USA); mouse monoclonal anti-β-actin (clone C4/actin, 1:500) from Becton Dickinson (Franklin Lakes, NJ, USA); rabbit polyclonal anti-CD69 (GTX37447, 1:100) from GeneTex, Inc. (Irvine, CA, USA); rabbit polyclonal anti-Sox5 (13216-1-AP, 1:1,000) and rabbit polyclonal anti-Sox6 (14610-1-AP, 1:1,000) from Proteintech (Rosemont, IL, USA); peroxidase-conjugated secondary antibodies (1:1,000) from GE Healthcare (Fairfield, CT, USA); and fluorescence-labelled secondary antibodies (488- or 594-conjugated, 1:1,000) from Life Technologies (Carlsbad, CA, USA).

### Primers

DNA primers were synthesized by Fasmac DNA synthesis service (Kanagawa, Japan). The primers used were 5′-ATGCCTGTGAAGATGGTCGCGATC-3′ (sense) and 5′-TCATCTACAAAATCCTGTTTCTTCATGAGACGGTC-3′ (antisense) for VCAM1; 5′-ATGGCTGCGGAAGCGATGTGC-3′ (sense) and 5′-CATGCCATAGCAAACACCAGTGG-3′ (antisense) for α4 integrin; 5′-ATGAATTCTGAAGAGTGTTCCATAACAGAAAATAGCTC-3′ (sense) and 5′-TCATAGGGAGGCCTTGCTGCAG-3′ (antisense) for CD69. The control β-actin primers were 5′-ATGGATGACGATATCGCTGCGCTC-3′ (sense) and 5′-CTAGAAGCATTTGCGGTGCACGATG-3′ (antisense).

### RT-PCRs

Total RNA was extracted using the Isogen kit (Nippon Gene, Tokyo, Japan). The cDNAs were prepared from 1 μg of total RNA with the Superscript reverse transcriptase kit (Life Technologies). PCR amplification was performed using the ExTaq DNA polymerase (Takara Bio, Kyoto, Japan) with 35 cycles, each consisting of denaturation at 94 °C for 1 min, annealing at 55–65 °C (depending on each primer’s Tm value) for 1 min, and extension at 72 °C for 1 min.

### Genetically modified/unmodified mice

Mice carrying the floxed allele of VCAM1 (JAX No. 007665), α4 integrin knockin mice harbouring the Arg-1007-to-Ala mutation whose mutant exhibits the biologically defective α4 integrin phenotype (JAX No. 010501), chondroitin sulfate proteoglycan4 (also called NG2) promoter-driven Cre recombinase transgenic mice (JAX No. 008533), and TAM-inducible PLP1 promoter-driven Cre recombinase transgenic mice (JAX No. 005975) were obtained from Jackson Laboratory (Bar Harbor, ME, USA). MBP promoter-driven Cre recombinase transgenic mice (RBRC No. 01461) were obtained from Riken BioResource Center (Ibaraki, Japan). MBP-Cre mice were mated with wild-type C57BL/6J mice for more than seven generations to achieve the same background as the other genetically modified mice. Genomic PCRs were performed according to established standard protocols (Jackson Laboratory or Riken BioResource Center). The primer pair for VCAM1 mutant mice was 5′-GGGACGGATTTTCTTTCCAC-3′ and 5′-GACTTTGAAGCCCATTGCAC-3′. The mutant allele consisted of ∼230 b and the wild type of ∼160 b. The primer pair for α4 integrin mutant mice was 5′-CAAGAGTCCGTTTGGGAAAA-3′ and 5′-CATGGCTTCTGCTTCTGC-3′. The mutant allele consisted of ∼315 b and the wild type of ∼215 b. The primer pair for Cre recombinase transgenic mice was 5′-CCACCACCTCTCCATTGCAC-3′ and 5′-ATGTTTAGCTGGCCCAAATG-3′. The transgenic allele consisted of ∼500 b. PCR was performed in 35 cycles, each consisting of denaturation at 94 °C for 1 min, annealing at 50–60 °C (depending on each primer’s Tm value) for 1 min, and extension at 72 °C for 1 min. The mutant mice were fertile under standard breeding conditions. Except in the case of NG2-Cre mice, male mice were used for experiments when their gender was distinguishable.

### Generation of CD69 transgenic mice

A DNA fragment (∼3.8 kb) containing all nucleotide units (SV40 enhancer, MBP promoter, CD69 and polyA^23^ was digested from the vector with *Sac*I and *Xho*I, purified, and injected into fertilized C57BL/6J oocytes^23^. Transgenic founder mice and established transgenic mice were identified both by genomic PCR (0.6 kb of each product) of tail DNA with specific primers (5′-CCGGAATTCGAATATTAGCTAGGAGTTTCAGAAAAGGGGGCCTG-3′ and 5′-CCGGAATTCACTAGTGGGACTATGGTTGCTGACTAATTGAGATGC-3′ for primers 1 and 2 and 5′-GATGACAAGAGCGGCATGAATTCTGAAGAGTGTTC-3′ and 5′-TCATAGGGAGGCCTTGCTGCAGATCC-3′ for primers 3 and 4) and by Southern blotting with *Stu*I-digested tail DNA hybridized to a radioisotope-labelled probe for an internal sequence of the transgene (∼0.6 kb of hybridized band). The *oct3/4* gene primers (5′-CCGGGATCCAAGCTTTGTGAACTTGGCGGCTTCCAAGTCG-3′ and 5′-CCGGGATCCCATTACTGGCCTGGTGCTTAGTTATCTTTG-3′) were used as the positive controls for genomic PCR (∼0.8 kb). PCR was performed in 35 cycles, each consisting of denaturation at 94 °C for 1 min, annealing at 55–65 °C (depending on each primer’s Tm value) for 1 min, and extension at 72 °C for 1 min. We succeeded in generating three founder mice, each harboring multiple transgenes, which were mated to wild-type C57BL/6J mice. In one transgenic line, the transgene was stably maintained for at least three generations. The transgenic mice were fertile and apparently normal in behaviour under standard breeding conditions. Male mice were used for experiments when their gender was distinguishable.

### Generation of CD69 shRNA transgenic mice

Mouse CD69 shRNA specifically, the target sequence 5′-TATACTGGTGCCATGGTCCTT-3′, was inserted into the BLOCK-iT PolII miR RNAi expression vector (Life Technologies), then amplified with 704–2,010 bases to isolate the fragment containing the CD69 shRNA sequence surrounded with the artificial miRNA backbone and the polyA signal sequence. MBP promoter^23^ and this fragment were successively inserted into the pCMV5 vector as the subcloning one. All nucleotide sequences were confirmed by Fasmac sequencing service. This knockdown construct is called shRNAmir (in this case, shCD69mir) (refs 24, 25). A DNA fragment (∼2.7 kb) containing all nucleotide units was digested from the vector with *Eco*RI and *Mlu*I, purified and injected into fertilized C57BL/6J oocytes. Transgenic founder mice and established transgenic mice were identified both by genomic PCR (∼0.8 kb of each product) of tail DNA with specific primers (5′-ATGGTGAGCAAGGGCGAGGAGCTG-3′ and 5′-CTTGTACAGCTCGTCCATGCCGAGAGTGATC-3′ for the middle region (primers 1 and 2) and 5′-GCTAACTGAAACACGGAAGGAGACAATACCGGAAG-3′ and 5′-CAGCTGCGCAGATCCATCAGAGATTTTGAGAC-3′ for the later region (primers 3 and 4)) and by Southern blotting with *Dra*I-digested tail DNA hybridized to a radioisotope-labelled probe for an internal sequence of the transgene (∼0.8 kb of hybridized band). PCR was performed in 35 cycles, each consisting of denaturation at 94 °C for 1 min, annealing at 55–65 °C (depending on each primer’s Tm value) for 1 min and extension at 72 °C for 1 min. In one transgenic line, the transgene was stably maintained for more than seven generations. The transgenic mice were fertile and apparently normal in behaviour under standard breeding conditions. Male mice were used for experiments when their gender was distinguishable.

### BrdU labelling *in vivo*

To investigate generation of new oligodendrocytes, we injected pregnant mice intraperitoneally with BrdU (100 mg kg^−1^ body weight; Sigma-Aldrich). About 3 weeks later, the perfused spinal cords of 16-day-old pups were dissected out and the cryosections were co-stained with anti-BrdU (Abcam) and anti-CC1 antibodies to visualize newly generated oligodendrocytes.

### TAM treatment *in vivo*

TAM (Sigma-Aldrich) was dissolved in a mixture of 90% corn oil and 10% ethanol at a concentration of 10 mg ml^−1^. Eight-week-old mice were given a daily intraperitoneal injection of 1 mg (100 μl) TAM for 5 consecutive days^42^. The first day of injections was designated day 0. Mice were killed at 28 days after TAM injection.

### Preparation of plasmids containing shRNAs

All nucleotide sequences were confirmed by sequencing. The sense and antisense oligonucleotides, which were used for duplex production, were as follows. VCAM1#1 (starting from nucleotide 231 of rat VCAM1) shRNA is composed of 5′-GATCCGTCCGTTCTGACCATGGACTTCAAGAGAGTCCATGGTCAGAACGGACTTTTTTACGCGTG-3′ and 5′-AATTCACGCGTAAAAAAGTCCGTTCTGACCATGGACTCTCTTGAAGTCCATGGTCAGAACGGACG-3′; VCAM1#2 (starting from nucleotide 393 of rat VCAM1) shRNA is composed of 5′-GATCCGCCGGTCATGGTCAAGTGTTTCAAGAGAACACTTGACCATGACCGGCTTTTTTTACGCGTG-3′ and 5′-AATTCACGCGTAAAAAAGCCGGTCATGGTCAAGTGTTCTCTTGAAACACTTGACCATGACCGGCG-3′; CD69 (starting from nucleotide 103 of rat CD69) shRNA is composed of 5′-GATCCGGATCCATTCAAGTTCCTATTCAAGAGATAGGAACTTGAATGGATCCTTTTTTTACGCGTG-3′ and 5′-AATTCACGCGTAAAAAAGGATCCATTCAAGTTCCTATCTCTTGAATAGGAACTTGAATGGATCCG-3′; and Sox6 (starting from nucleotide 80 of rat Sox6) shRNA is composed of 5′-GATCCGGGAGGAGGAAGAGGGTAGCTGTGAAGCCACAGATGGGCTGCCCTCTTCCTTTTCCCTTTTTTACGCGTAT and 5′-CGATACGCGTAAAAAAGGGAAAAGGAAGAGGGCAGCCCATCTGTGGCTTCACAGCTACCCTCTTCCTCCTCCCG. The control *Photinus pyralis* luciferase shRNA is composed of 5′-GATCCGGCCATTCTATCCTCTAGAGTTCAAGAGACTCTAGAGGATAGAATGGCCTTTTTTAGATCTC-3′ and 5′-AATTCAGATCTAAAAAAGGCCATTCTATCCTCTAGAGTCTCTTGAACTCTAGAGGATAGAATGGCCG-3′. The annealed duplexes were ligated into a retrovirus vector, namely, either pSINmU6 (Takara Bio) or pSIREN-RetroQ-ZsGreen1 (Takara Bio).

### Plasmids’ construction

VCAM1 or CD69 cDNA was amplified from human brain total RNA by RT-PCR. Each coding region was confirmed by sequencing and finally inserted into the retrovirus vector pMEI5 (Takara Bio).

### Primary oligodendrocyte cultures

Oligodendrocyte precursor cells were isolated from embryonic day 15 (E15) Sprague–Dawley rats^43^,^44^,^45^,^46^. Briefly, cerebral cortices were dissected, dissociated with 0.25% trypsin, triturated and passed through mesh with 70 μm pores. Cells were collected, resuspended in MEM containing 10% fetal bovine serum, 50 U ml^−1^ penicillin and 50 μg ml^−1^ streptomycin, and seeded on poly-L-lysine-coated dishes. After two passages, the cells were cultured on non-coated Petri dishes (Thermo Scientific, Waltham, MA, USA). In some experiments, recombinant α4β1 integrin-coated dishes were used (R&D Systems). On the second day of culture, the medium was changed to a DMEM-based serum-free growth medium containing growth factors PDGF and FGF (PDGF-AA and bFGF; Peprotech, Rocky Hill, NJ, USA) and N2 (Life Technologies) and cells were cultured for an additional 2 days. These cells were then used as oligodendrocyte precursor cells (a proliferating state before differentiation). To induce their differentiation, cells were cultured with a differentiation medium containing 20 ng ml^−1^ triodothyronine and 20 ng ml^−1^ thyroxine but no growth factor. After 3 days, these cells were gradually differentiated into mature oligodendrocytes with MBP-positive myelin web-like structures. To confirm viability of attached cells, cells were stained with 0.4% trypan blue. Trypan blue-incorporating cells numbered fewer than 5% in each experiment in this study.

### Production of retroviruses and retrovirus-mediated DNA transfection

Using the CalPhos Transfection kit (Takara Bio), each retrovirus vector was co-transfected into G3Thi cells with vectors pVSVG and pGP, which were provided with the Takara Bio retroviral production kit. After 2 days, each culture supernatant was centrifuged at 10 000 r.p.m. for 8 h to concentrate the recombinant retroviruses. The virus pellets were suspended in each culture medium. Since retroviruses are infected into proliferating cells, they were used for transfection (infection) into growing, primary oligodendrocyte precursor cells before being co-cultured with neurons^43^,^44^.

### Oligodendrocyte-neuronal co-cultures

DRG neurons were isolated from E15 SD rats, then dissociated and plated onto collagen (type I)-coated 22-mm-coverslips^47^,^48^,^49^. Non-neuronal growing cells were eliminated by cycling three times with medium containing 5-fluorodeoxyuridine and uridine. Co-cultures were established by seeding ∼200,000 purified oligodendrocyte precursor cells on neurons. Co-cultures were maintained in the presence or absence of a recombinant VCAM1-Fc protein (R&D Systems) or an antibody for 3 to 4 weeks. Medium was replaced every 3 to 4 days. For quantification of attachment and alignment of oligodendrocytes to axons, oligodendrocytes with attached processes were defined as those with processes <20 μm long attached to axons. Oligodendrocytes with aligning processes, meanwhile, were defined as those with processes >20 μm long attached to axons. The percentages of positive cells for each marker were determined blindly from >3 fields.

### Immunoblotting

Tissues or cells were lysed in lysis buffer (50 mM HEPES-NaOH (pH 7.5), 20 mM MgCl_2_, 150 mM NaCl, 1 mM dithiothreitol, 1 mM phenylmethane sulfonylfluoride, 1 μg ml^−1^ leupeptin, 1 mM EDTA, 1 mM Na_3_VO_4_, 10 mM NaF, 0.5% NP-40, 1% CHAPS and 0.1 or 0.3% SDS) to elute myelin segment proteins and were centrifuged in a microcentrifuge to obtain clear supernatants^47^,^48^,^49^. These lysates (10 μg per sample on average) were denatured and subjected into SDS-polyacrylamide gels. The electrophoretically separated proteins were transferred to a PVDF membrane, blocked with Blocking One reagent (Nacalai Tesque, Kyoto, Japan), and immunoblotted first with primary antibodies and then with peroxidase-conjugated secondary antibodies. The bound antibodies were detected using a normal- or high-sensitivity chemiluminescence detection kit (Nacalai Tesque). At least three experiments were carried out under each condition and representative blots are shown in the figures. Images have been cropped for presentation. Full-size images are presented in Supplementary Figs 18 and 19.

### Immunofluorescence

Cells on coverslips or dishes were normally fixed with 4% paraformaldehyde (PFA). In all, 4% PFA followed by 100% cold methanol was used for myelin segment staining^47^,^48^,^49^. Cells were permeabilized with phosphate-buffered saline (PBS) containing 0.1% Tween-20 or 0.1% Triton X-100, blocked with Blocking One reagent, and incubated first with primary antibodies and then with fluorescence-labelled secondary antibodies. The coverslips or dishes were mounted with Vectashield reagent with DAPI (Vector Laboratories, Burlingame, CA, USA). The fluorescence images were captured with a DMI4000B fluorescence microscope system (Leica, Wetzlar, Germany) and analysed with AF6000 software (Leica). At least three experiments were carried out under each condition and representative photographs are shown in the figures.

### Immunohistochemistry

Tissues were perfused first with PBS, and then with PBS containing 4% PFA. The tissues were postfixed with 4% PFA, replaced with 20% sucrose and embedded in Tissue-Tek reagent (Sakura Finetechnical, Tokyo, Japan)^47^,^48^,^49^. The cryosections for immunostaining with an antibody for O4 were prepared according to the manufacturer’s instructions (Merck-Millipore). Microtome sections on glass slides were blocked with Blocking One reagent, and incubated first with primary antibodies and then with fluorescence-labelled secondary antibodies. The glass slides were mounted with Vectashield reagent. The fluorescent images were collected with an IX81 microscope system (Olympus, Tokyo, Japan) equipped with a laser-scanning FV500 or FV1000D (Olympus) and analysed with Fluoview software (Olympus). At least three experiments were carried out under each condition and representative photographs are shown in the figures. The percentages of positive cells for each marker were determined blindly from >3 fields.

### Electron microscopy

Tissues were fixed with 2% PFA and 2% glutaraldehyde in 0.1% cacodylate-containing buffer. They were then contrasted with 2% osmium tetroxide, dehydrated with an ethanol gradient, and treated with propylene oxide. Finally, samples were infiltrated and embedded in pure epoxy resin. Ultrathin sections were stained with uranyl acetate and lead staining solution. Images were taken using a JEM-1200EX electron microscope system (JOEL, Tokyo, Japan) by Hana-ichi Ultrastructure Research Institute (Nagoya, Japan).

### Gene microarray analysis

Total RNA was labelled with Cy5 using the Amino Allyl MessageAMP II aRNA Amplification Kit (Life Technologies). A 3D-Gene chip (rat oligo chip 20k; Toray, Tokyo, Japan) was used for microarray analysis by Toray 3D-Gene chip service. The signals, which were hybridized to Cy5-labelled aRNA pools, were obtained using a Toray 3D-Gene scanner. The detected signals for the respective genes were normalized according to the global normalization method and were processed by Toray 3D-Gene analytical service^50^. The median value of the detected signal intensities was adjusted to 25.

### Statistical analysis

Experiments were not randomized. Data collection and analysis were performed blindly to the conditions of the experiments. No data were excluded from the analyses. Values shown in figure panels represent the means±s.d. from separate experiments. Comparisons between two experimental groups were made using Student’s *t*-test (**P<0.01, *P<0.05). A one-way analysis of variance was followed by a Fisher’s protected least significant difference test as a *post hoc* comparison (**P<0.01). No statistical methods were used to predetermine sample sizes. The sample sizes are similar to those used in the field.

### Ethics statement

Genetically modified/unmodified mice were dealt with in accordance with a protocol approved by the Japanese National Research Institute for Child Health and Development Animal Care Committee. Gene recombination processes were dealt with in accordance with a protocol approved by the Japanese National Research Institute for Child Health and Development Gene Recombination Committee.

### Data availability

Microarray data have been deposited in the Gene Expression Omnibus (GEO) database under accession code GSE86862. The authors declare that the data supporting the findings of this study are available within the article and its Supplementary Information Files or from the corresponding authors on reasonable request.

## Additional information

**How to cite this article:** Miyamoto, Y. *et al*. VCAM1 acts in parallel with CD69 and is required for the initiation of oligodendrocyte myelination. *Nat. Commun.* **7,** 13478 doi: 10.1038/ncomms13478 (2016).

**Publisher’s note**: Springer Nature remains neutral with regard to jurisdictional claims in published maps and institutional affiliations.

## Supplementary Material

Supplementary Information Supplementary Figures 1 - 19 and Supplementary Tables 1 - 2
