# Supplementary material for: VCAM1 acts in parallel with CD69 and is required for the initiation of oligodendrocyte myelination
Source: Nat Commun. 2016 Nov 23;7:13478. doi: 10.1038/ncomms13478 (PMC5476804; doi:10.1038/ncomms13478)
Supplement: Supplementary Information — Supplementary Figures 1 - 19 and Supplementary Tables 1 - 2 [file ncomms13478-s1.pdf]

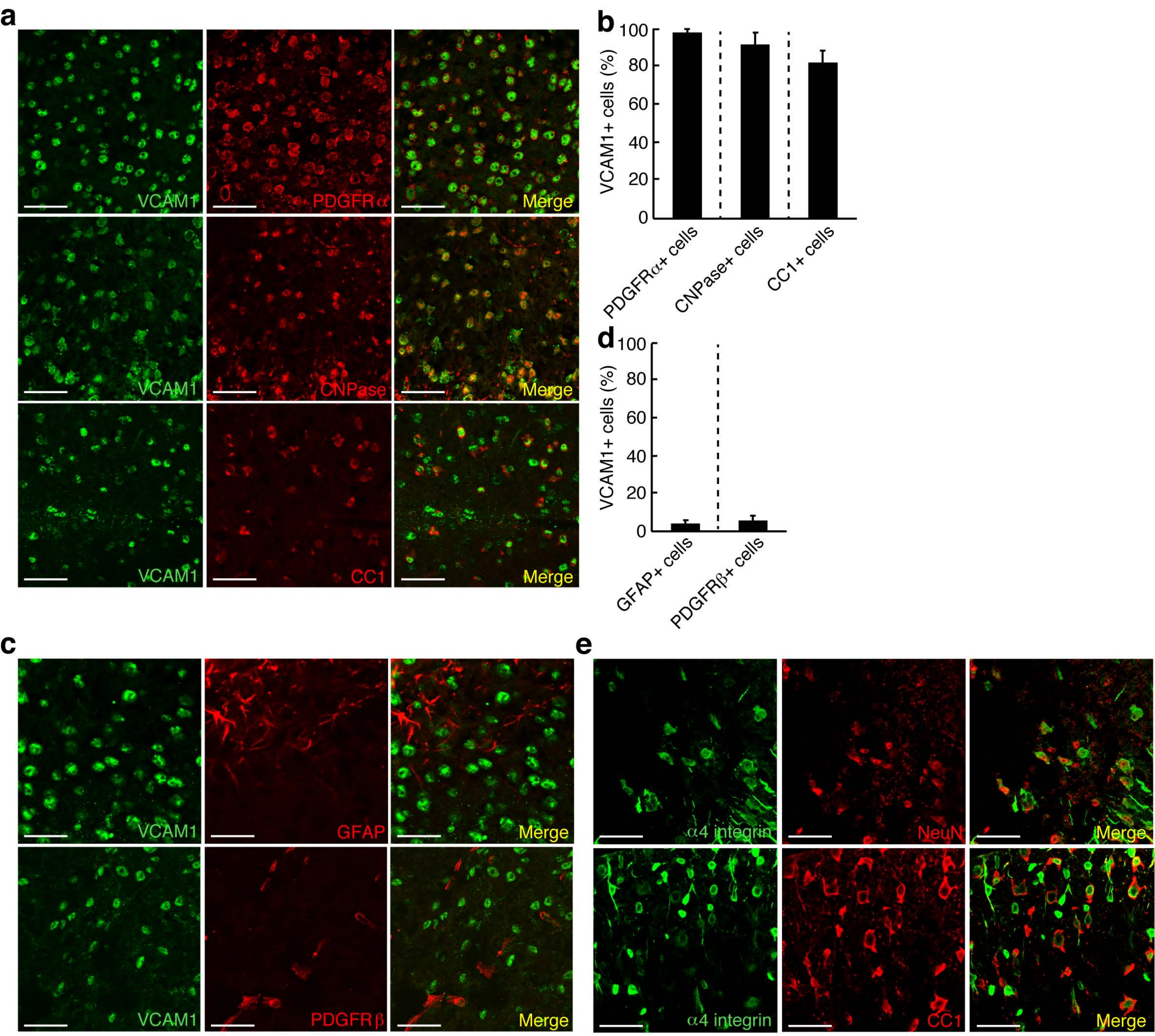

Supplementary Fig. 1. Expression of VCAM1 and α4 integrin proteins in the CNS. (a, b) Cross sections of 3-, 14-, or 21-day-old mouse spinal cords were costained with antibodies against VCAM1 (green) and PDGFRα, CNPase, or CC1 (red), respectively. Data are representative. The scale bars indicate 50 μm. The percentage of VCAM1+ cells among the PDGFRα+, CNPase+, or CC1+ cells is shown (n=10-32 slices for three independent experiments). (c, d) Cross sections of 7-day-old mouse spinal cords were costained with antibodies against VCAM1 (green) and GFAP or PDGFRβ (red). Data are representative. The scale bars indicate 50 μm. The percentage of GFAP+ or PDGFRβ+ cells among the VCAM1+ cells is shown (n=12-15 slices for two independent experiments). (e) Spinal cord sections at postnatal day 7 were costained with antibodies against α4 integrin (green) and CC1 or NeuN (red).Data are representative. The scale bars indicate 50 μm.

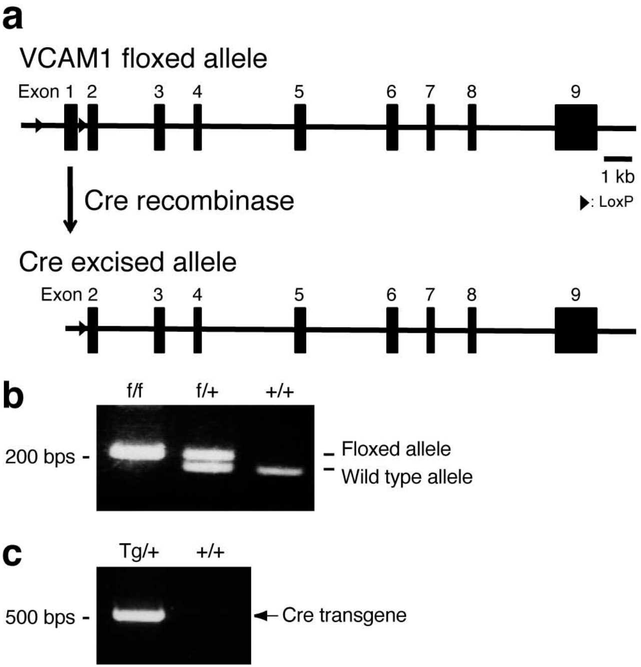

Supplementary Fig. 2. Identification of the conditional VCAM1 knockout mouse. (a) Schematic diagram of the Cre recombinase-mediated VCAM1 conditional knockout allele. (b) Confirmation of the VCAM1 floxed alleles (f/f, f/+, or +/+) by genomic PCR (representative data). (c) Confirmation of presence or absence of the Ng2-Cre transgene (Tg) by genomic PCR (representative data).

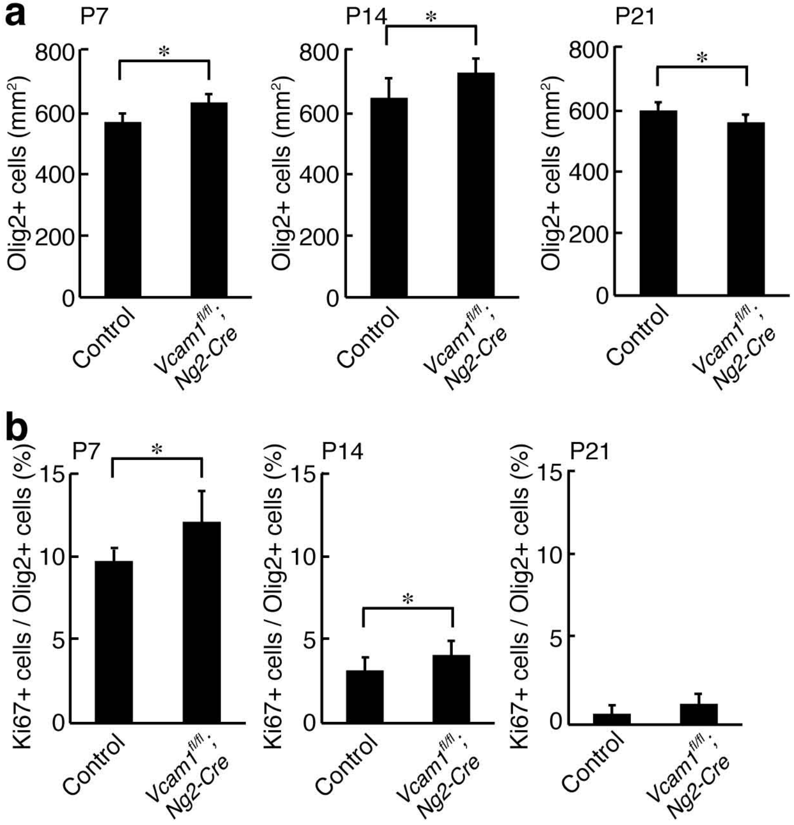

Supplementary Fig. 3. Knockout of VCAM1 increases proliferating oligodendrocyte lineage cells. (a, b) Spinal cord sections at postnatal days 7, 14, and 21 were costained with antibodies against Ki67 and/or Olig2. The number of Olig2+ cells per one square millimeter was counted. Data were evaluated using Student's *t* test (*, p=0.0167 (P7), p=0.0193 (P14), or p=0.0399 (P21); n=5-11 slices of two independent experiments). The percentage of Ki67+ cells among the Olig2+ cells is shown. Data were evaluated using Student's *t* test (*, p=0.0495 (P7), p=0.0249 (P14), or non-significance (P21); n=5-10 slices of two independent experiments).

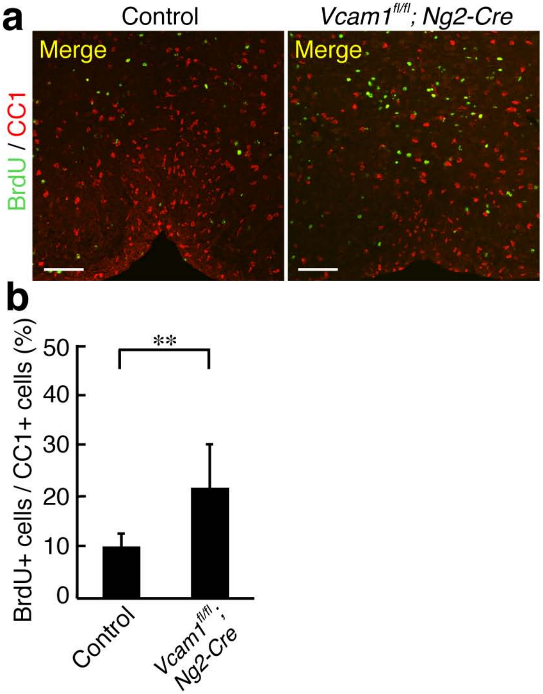

Supplementary Fig. 4. Knockout of VCAM1 results in decreased BrdU incorporation.(a, b) Pregnant mice were intraperitoneally injected with BrdU. About three weeks later, the spinal cords of 16-day-old pups were dissected out and costained with antibodies against BrdU (green) and CC1 (red). Data are representative. The scale bars indicate 200 µm. The percentage of BrdU+cells among the CC1+ cells is shown. Data were evaluated using Student' s *t* test (**, *p*=0.000181; n=10 slices of two independent experiments).

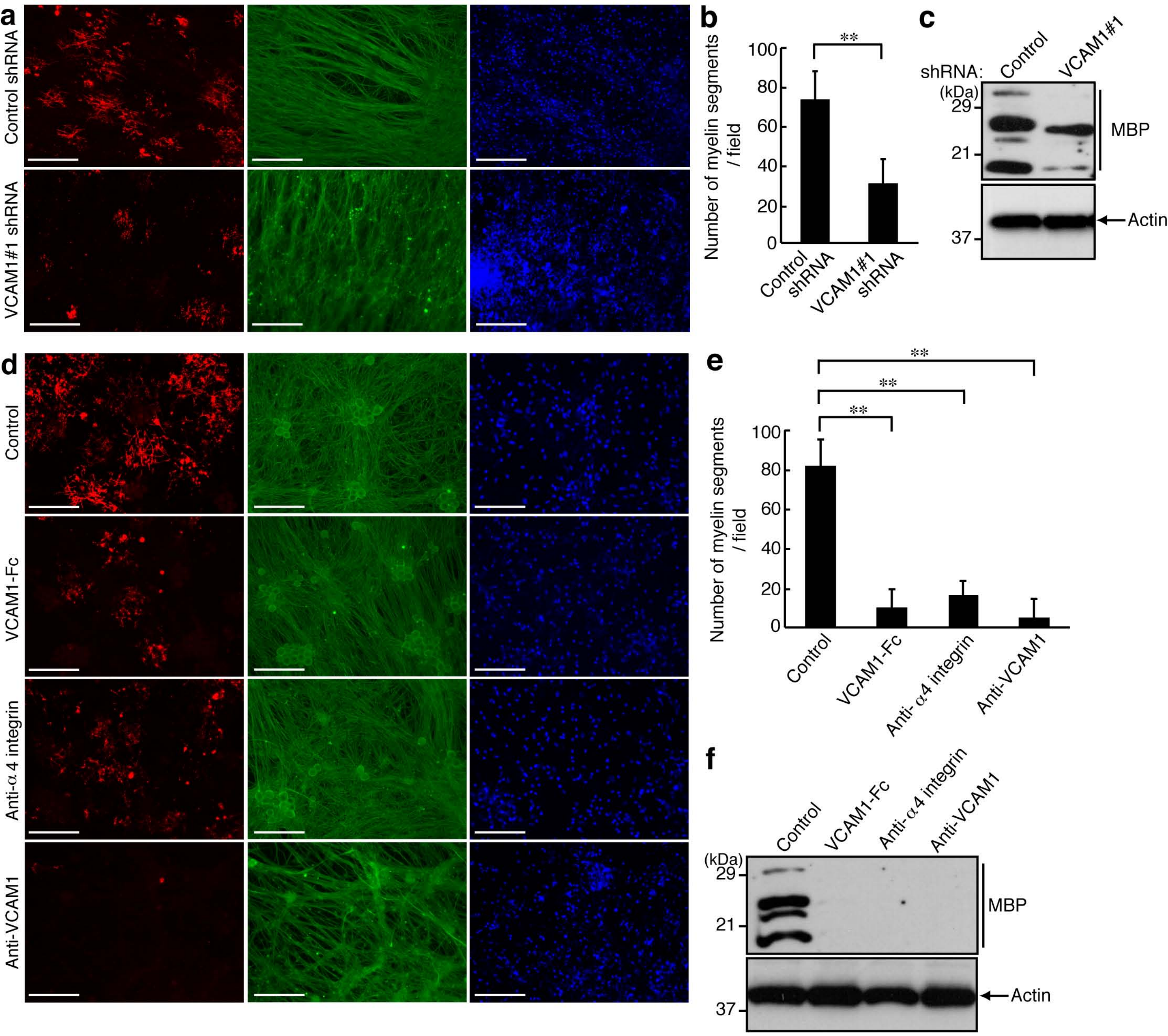

Supplementary Fig. 5. Inhibition of VCAM1 or its interaction decreases myelin formation in cocultures. (a, b) Oligodendrocytes that had been transfected with VCAM1 shRNA (VCAM1#1) or control were cocultured with neurons, then costained with antibodies against MBP (red), NF (green) and DAPI (blue), in order to detectmyelin segments in ROIs. Data are representative. The scale bars indicate 200 µm. Data were evaluated using Student' s *t* test (**, *p*=4.65E-06; n=10-15 areas of three independent experiments). (c) The lysates were immunoblotted with an antibody against MBP or actin. Data are representative of three experiments. (d, e) Cocultures were performed in the presence or absence of VCAM-Fc or the respective antibodies against α4 integrin and VCAM1, and staining for MBP (red), NF (green), and DAPI (blue) was performed. Data are representative. The scale bars indicate 100 µm. Data were evaluated using Student' s *t* test (**, *p*=5.10E-13 (VCAM1-Fc), 5.49E-15(anti-α4 integrin), or 5.36E-17 (anti-VCAM1); n=13-20 areas of three independent experiments). (f) The lysates were immunoblotted with an antibody against MBP or actin. Data are representative of two independent experiments.

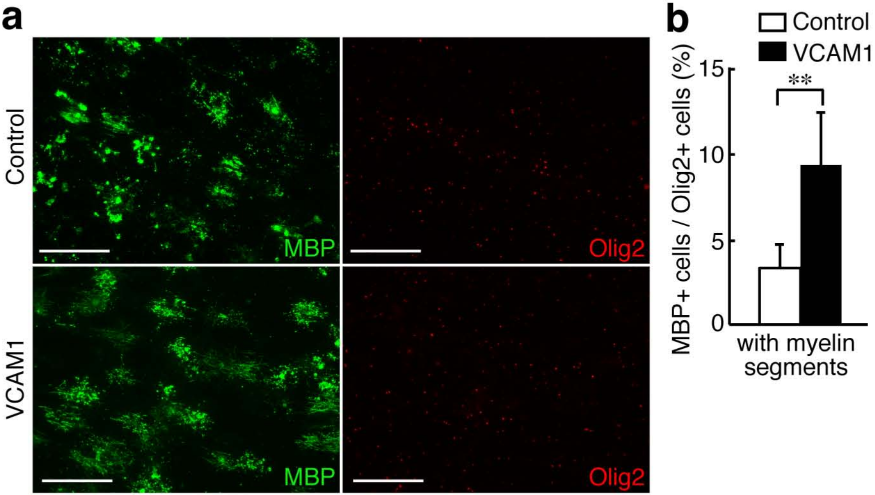

Supplementary Fig. 6. Transfection of VCAM1 promotes myelin formation in cocultures. (a) Oligodendrocytes that had been transfected with the plasmid encoding VCAM1 or control mock were cocultured with neurons in a growth medium containing PDGF and bFGF and costained with antibodies against MBP (green) for Olig2 (red). Data are representative.The scale bars indicate 200 μm. (b) The percentage of MBP+ cells with myelin segments among the Olig2+ cells is shown. Data were evaluated using Student' s *t* test (**, *p*=2.87E-10; n=20-27 areas of two independent experiments).

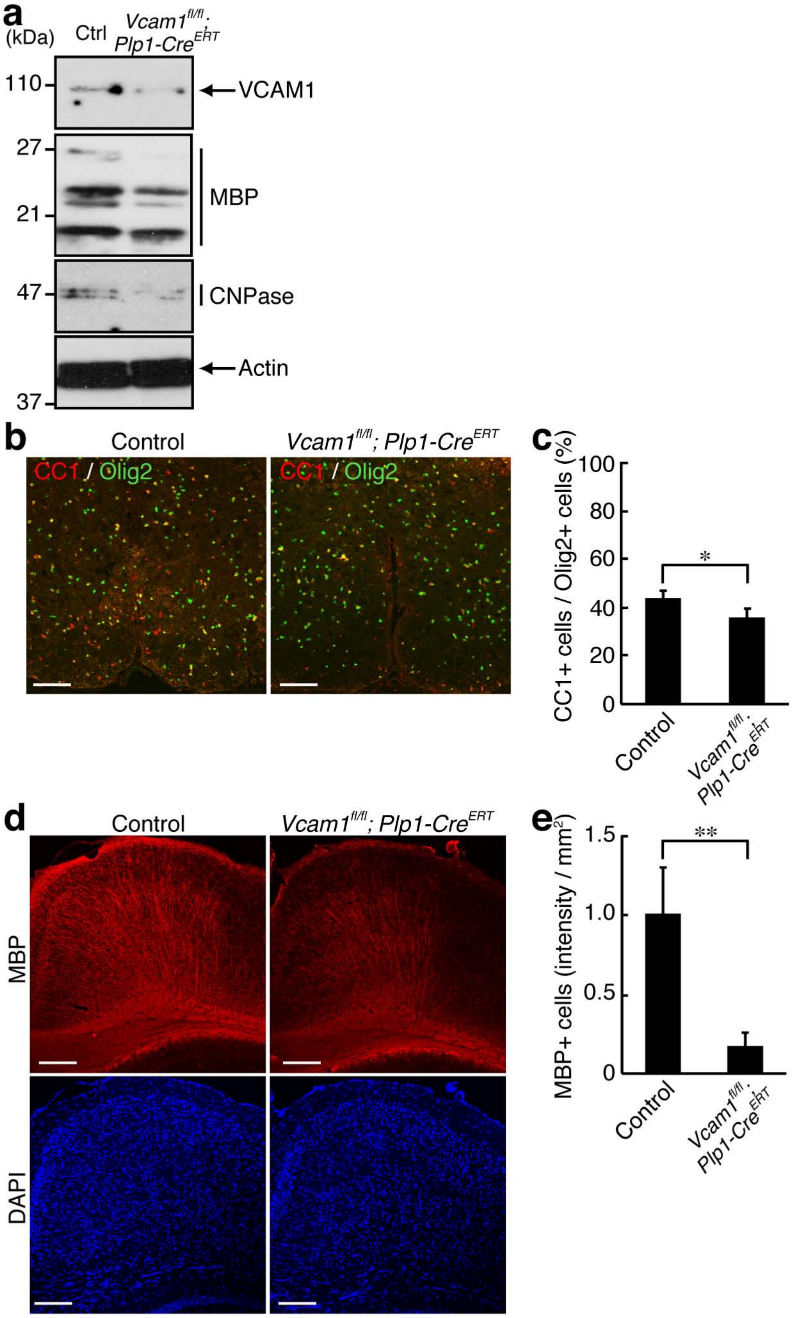

Supplementary Fig. 7. Knockout of VCAM1 decreases myelin marker protein expression in adult mice. (a) 8-week-old mice were given a daily intraperitoneal injection of 1 mg (100µl) TAM or 5 consecutive days. 23 days following the final TAM injection, lysates from TAM-treated PLP1-CreERT-driven VCAM1 conditional knockout (VCAM1$^{fl/fl}$; PLP1-CreERT) or control (Ctrl) mouse spinal cords were immunoblotted with an antibody against VCAM1, MBP, CNPase, or actin. Data are representative of two experiments. (b, c) Antibodies against CC1 (red) and Olig2 (green) were used for costaining in TAM-treated VCAM1$^{fl/fl}$; PLP1-CreERT or control mouse spinal cord cross sections. The scale bars indicate 100 µm. The percentage of CC1+ cells among the Olig2+ cells is shown. Data were evaluated using Student' s *t* test (*, *p*=0.0127; n=9 slices of two independent experiments). (d, e) Corpus callosum sections of VCAM1$^{fl/fl}$; PLP1-CreERT and control mouse were costained with an anti-MBP antibody (red) and DAPI (blue). The scale bars indicate 200 µm. The intensity of MBP staining per one square millimeter was semi-quantified. Data were evaluated using Student' s *t* test (*, *p*=0.0178; n=4 slices of two independent experiments).

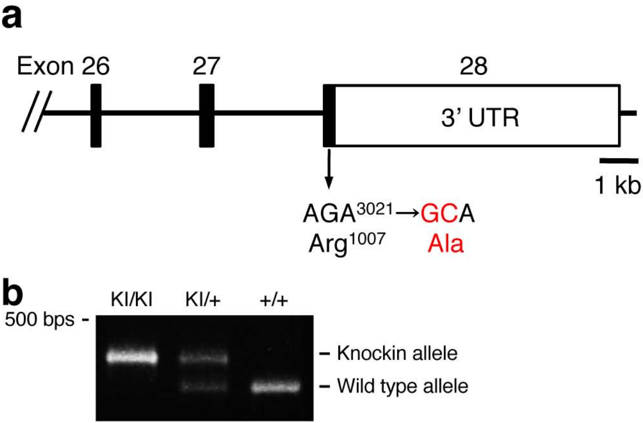

Supplementary Fig. 8. Identification of the α4 integrin knockin mouse. (a) Schematic diagram of the mouse α4 integrin knockin (Arg-1007-to-Ala) allele. (b) Confirmation of the knockin alleles (KI/KI, KI/+, or +/+) by genomic PCR (representative data).

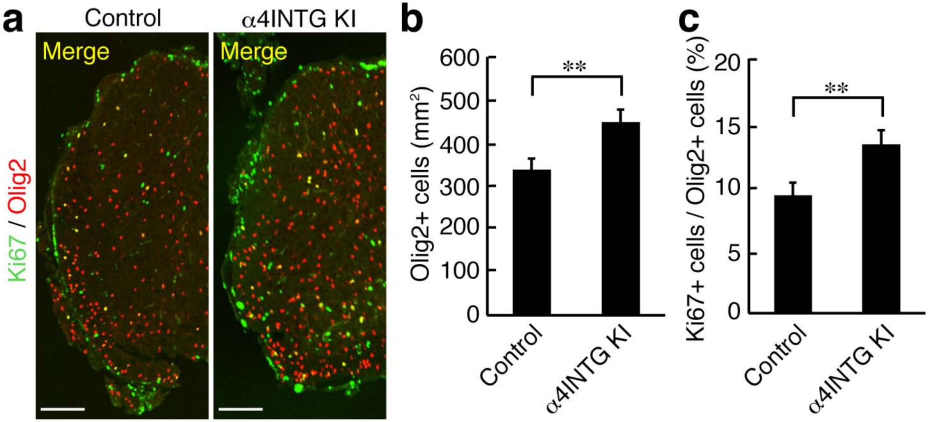

Supplementary Fig. 9. Mutant mice of α4 integrin exhibit increased proliferating oligodendrocyte lineage cells. (a-c) Antibodies against Ki67 (green) and Olig2 (red) were used for costaining in 2-day-old α4 integrin knockin (α4INTG KI) or control mouse spinal cord cross sections. Data are representative. The scale bars indicate 200 μm. The number of Olig2+ cells per one square millimeter was counted. Data were evaluated using Student' s *t* test (**, p=1.27E-05; n=6-10 slices of two independent experiments). The percentage of Ki67+ cells among the Olig2+ cells is shown. Data were evaluated using Student' s *t* test (**, p=1.66E-05; n=6-11 slices of two independent experiments).

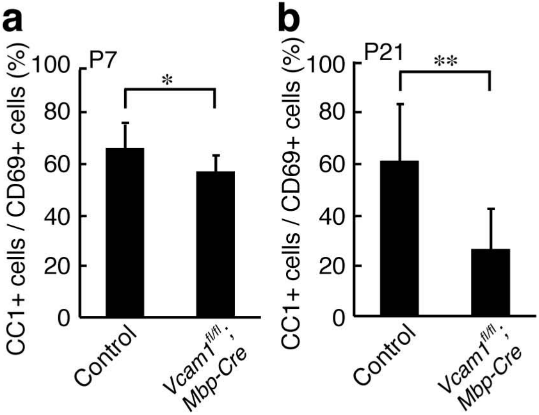

Supplementary Fig. 10. Expression of CD69 proteins in VCAM1 cKO mice.

(a, b) Cross sections of 7- and 21-day-old mouse spinal cords were costained with antibodies against CD69 and CC1. The percentage of CC1+ cells among the CD69+ cells is shown. Data were evaluated using Student’ s *t* test (**, *p*=0.00336 (P21) or *, *p*=0.0410 (P7) ; n=9-12 slices of two independent experiments).

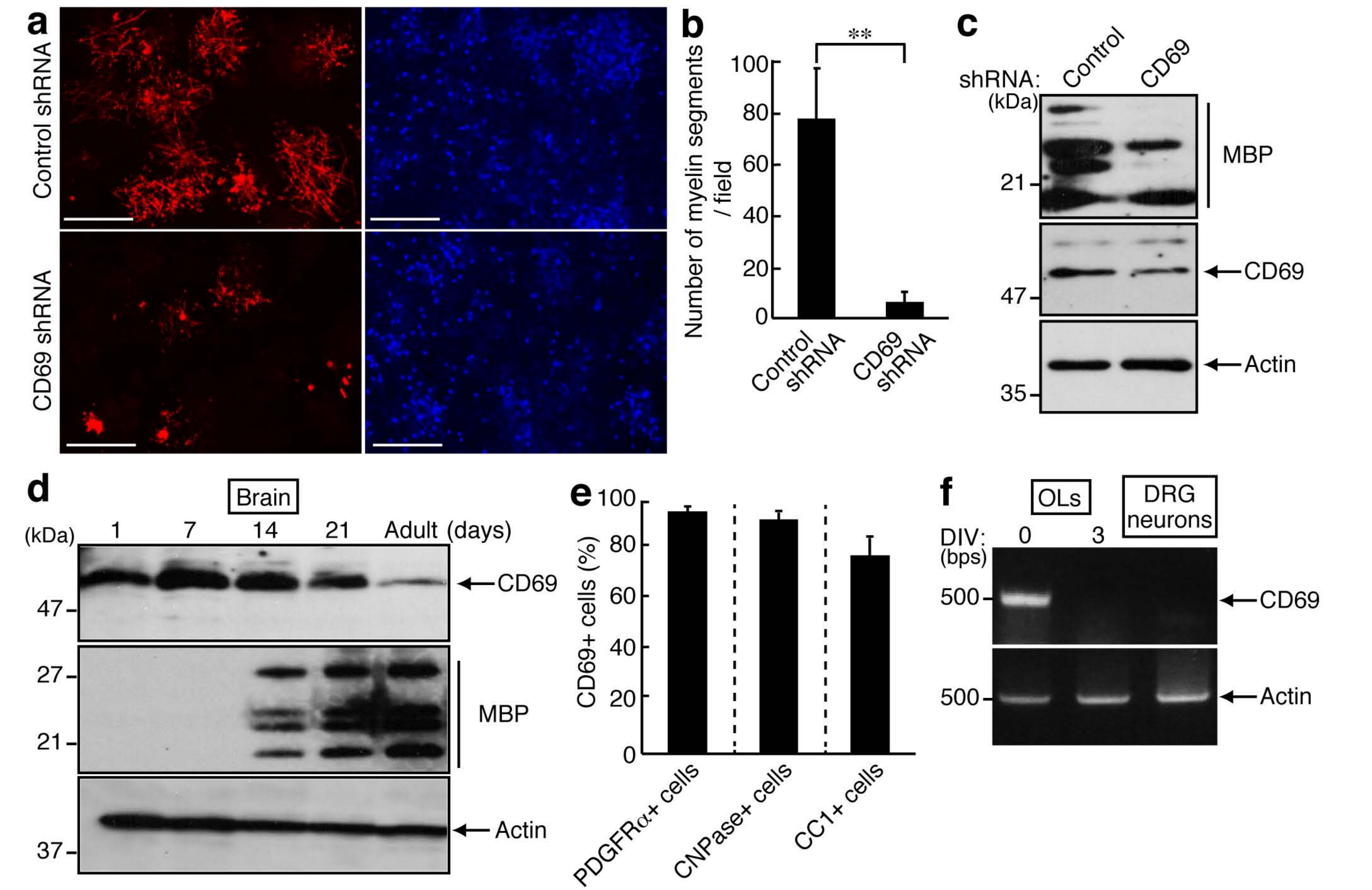

Supplementary Fig. 11. Knockdown of CD69 decreases myelin formation in cocultures. (a, b) Oligodendrocytes that had been transfected with CD69 shRNA or control were cocultured with neurons and costained with an anti-MBP antibody (red) and DAPI (blue). Data are representative. The scale bars indicate 100 µm. Data were evaluated using Student' s *t* test (**, *p*=1.78E-28; n=31-35 areas of two independent experiments. (c) The lysates were immunoblotted with an antibody against MBP, CD69, or actin. Data are representative of two experiments. (d) Tissue extracts were prepared from mouse brains on postnatal days 1-21 or in adulthood and immunoblotted with an antibody against CD69, MBP, or actin. Data are representative of two experiments. (e) Cross sections of 3-, 14-, or 21-day-old mouse spinal cords were costained with antibodies against CD69 and PDGFRα, CNPase, or CC1, respectively.The percentage of CD69+ cells among the PDGFRα+, CNPase+, or CC1+ cells is shown (n=6-10 slices of two independent experiments). (f) RT-PCR analysis for CD69 and actin was performed in differentiating oligodendrocytes or neurons. Data are representative.

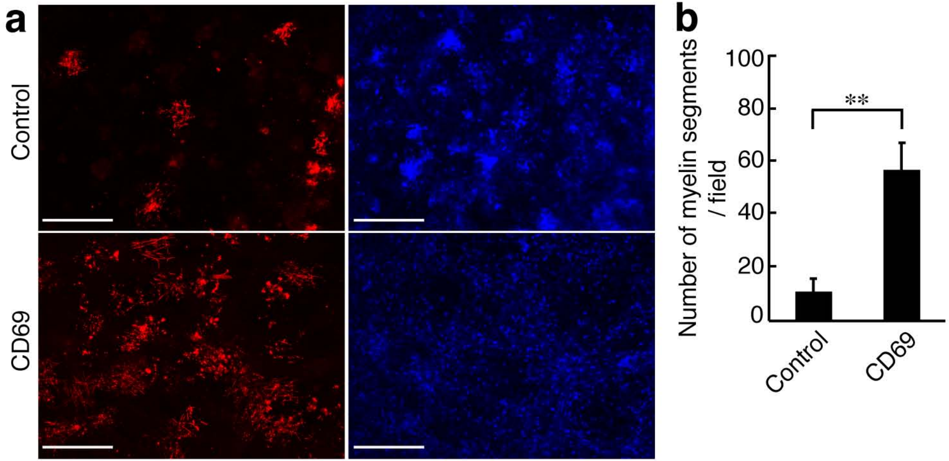

Supplementary Fig. 12. Transfection of CD69 can reverse the phenotype of VCAM1-knocked down cocultures. (a, b) Oligodendrocytes were transfected with the plasmid encoding CD69 or control together with VCAM1 shRNA (VCAM1#1) and cocultured with neurons. Cocultures were costained with an anti-MBP antibody (red) and DAPI (blue). Data are representative. The scale bar indicates 200 µm. Data were evaluated using Student' s *t* test (**, *p*=3.57E-16; n=17-18 areas of two independent experiments).

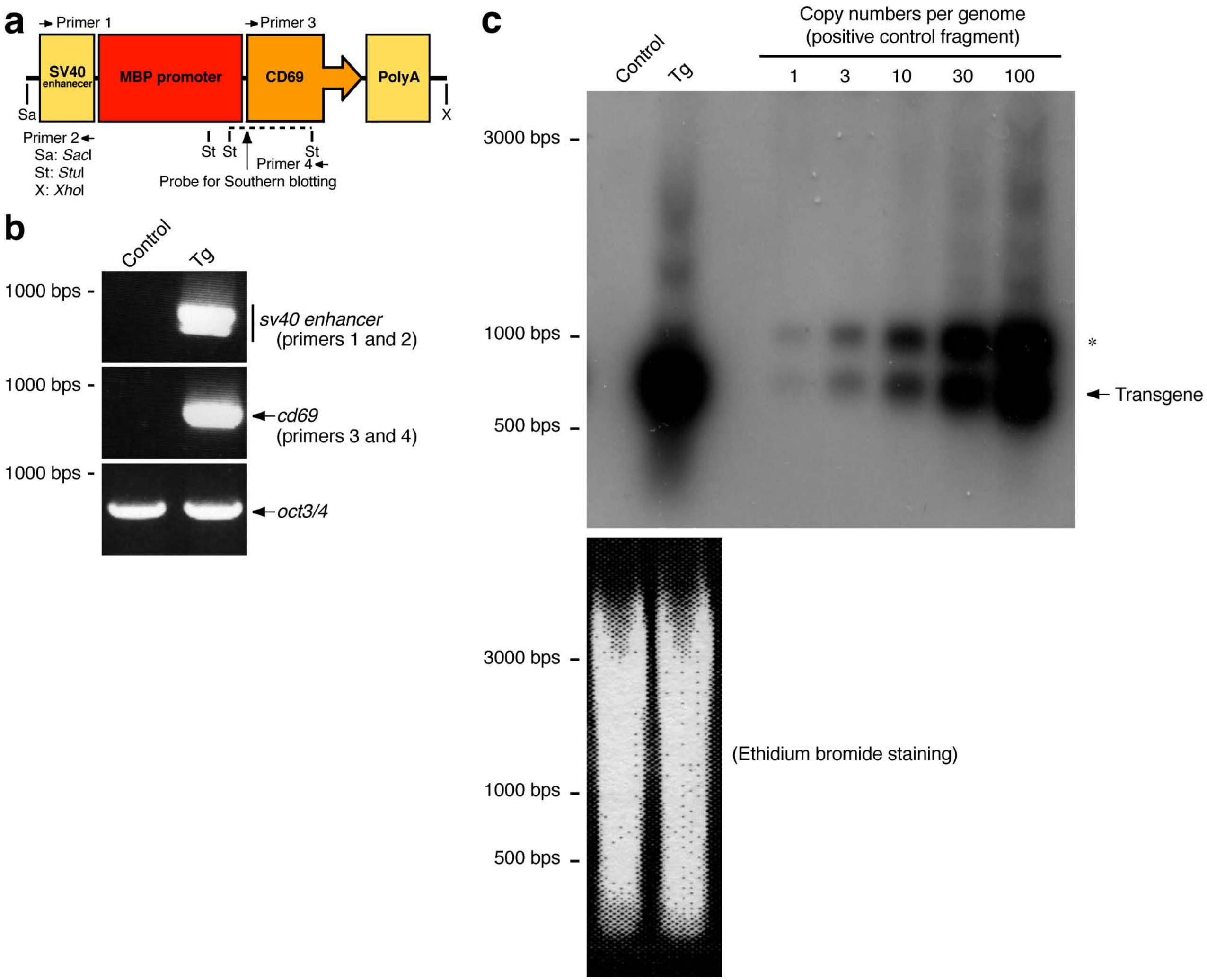

Supplementary Fig. 13. Generation of CD69 transgenic mice. (a) Schematic diagram of the CD69 transgene. The positions of genomic PCR primers (primer 1 to primer 4), Southern blotting probe, and major restriction enzyme recognition sites (single SacI site, three StuI sites, and single XhoI site) are shown. (b, c) To identify transgenic or nontransgenic mice (Tg or control), genomic PCR was performed (representative data). PCR for Oct3/4 is also shown as a control experiment. Southern blotting, using StuI-digested genome, was performed using a radioisotope-labeled transgene fragment as the probe. The copy numbers of transgenes were greater than 100; this lane was compared to positive control fragment lanes. The position of the asterisk corresponds to that of the first and third sites of StuI-digested transgene fragment. Ethidium bromide staining for total RNAis also shown.

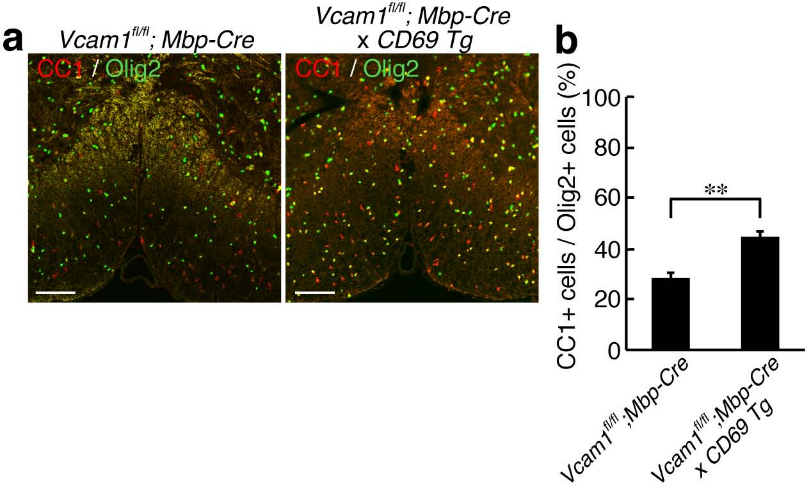

Supplementary Fig. 14. CD69 transgenic genotype in vivo can reverse the VCAM1 knockout phenotype. (a, b) CD69 transgenic (Tg) mice were crossbred with VCAM1$^{fl/fl}$; Mbp-Cre mice. Antibodies against CC1 (red) and Olig2 (green) were used for costaining in 8-day-old VCAM1$^{fl/fl}$; Mbp-Cre or VCAM1$^{fl/fl}$; Mbp-Cre x CD69 Tg mouse spinal cord cross sections. Data are representative. The scale bars indicate 100 µm. The percentage of CC1+ cells among the Olig2+ cells is shown. Data were evaluated using Student' s *t* test (**, p=2.27E-06; n=9 slices of two independent experiments).

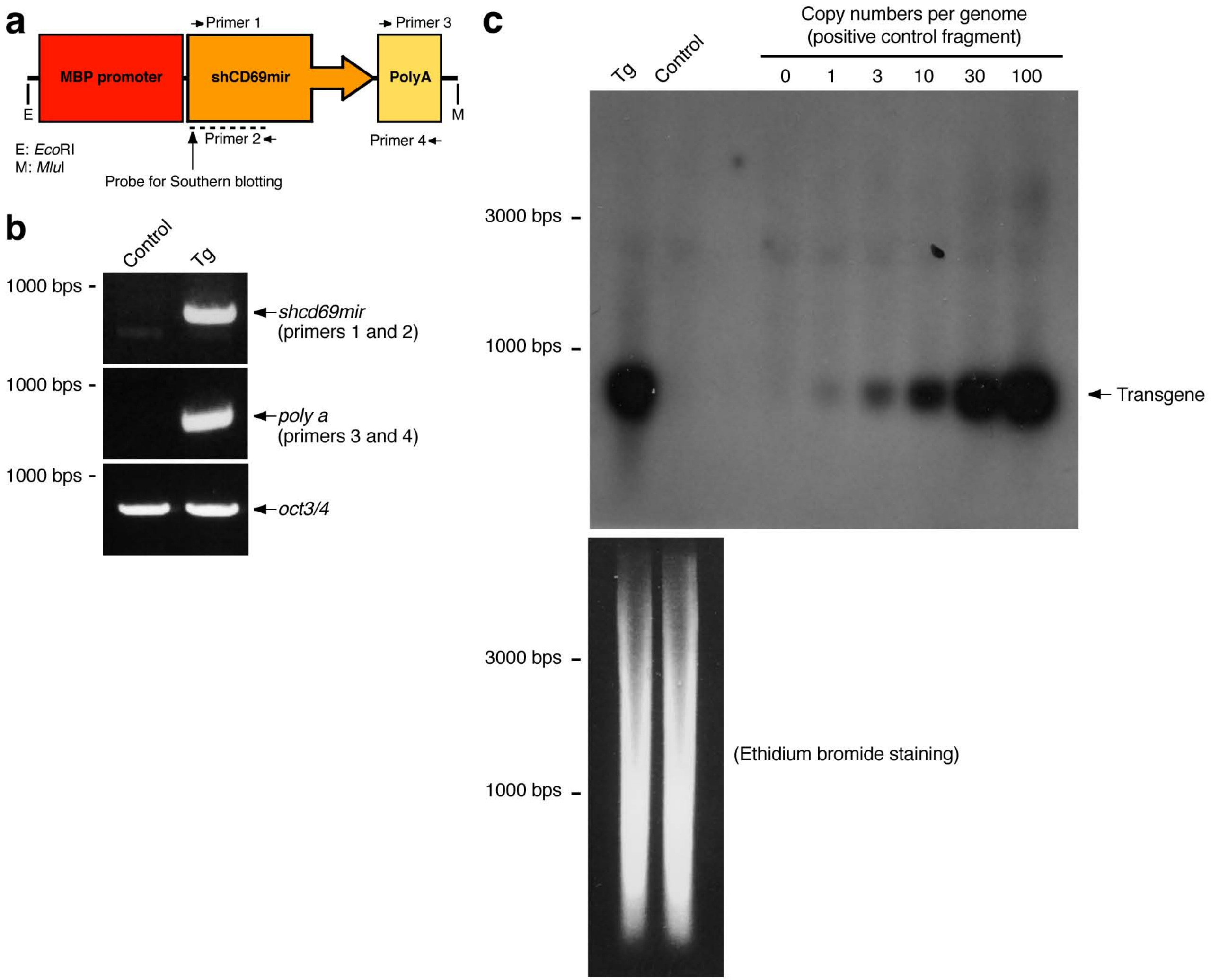

Supplementary Fig. 15. Generation of CD69 shRNA transgenic mice. (a) Schematic diagram of the CD69 shRNA transgene (often called shCD69mir). The positions of genomic PCR primers (primer 1 to primer 4), Southern blotting probe, and major restriction enzymes are shown. (b, c) Transgenic mice or nontransgenic controls (Tg or control) were identified by genomic PCR (representative data). PCR for Oct3/4 is also shown as a control experiment. Southern blotting, using DraI-digested genome, was performed using a radioisotope-labeled transgene fragment as the probe. The copy numbers of transgenes were roughly 75; this lane was compared to positive control fragment lanes. Ethidium bromide staining for total RNA is also shown.

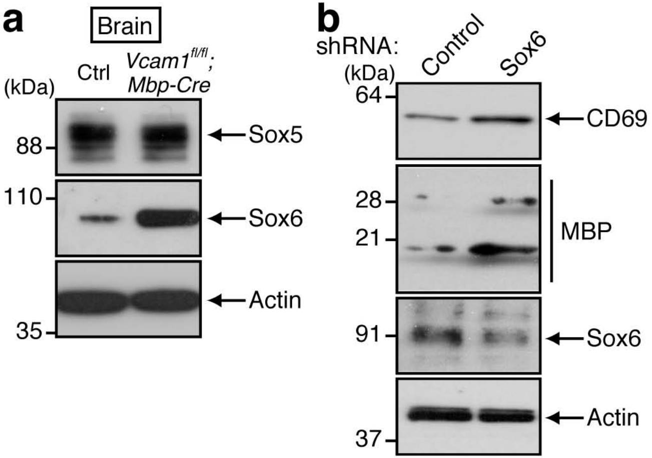

Supplementary Fig. 16. The role of Sox6, a protein upregulated by VCAM1 knockout, on CD69 expression. (a) Tissue lysates from 11-day-old VCAM1$^{fl/fl}$; Mbp-Cre or control mouse whole brains were immunoblotted with an antibody against Sox5, Sox6, or actin. Data are representative of three experiments. (b) Oligodendrocytes were transfected with Sox6 or control shRNA and cocultured with neurons. The lysates were immunoblotted with an antibody against CD69, MBP, Sox6, or actin. Data are representative of two experiments.

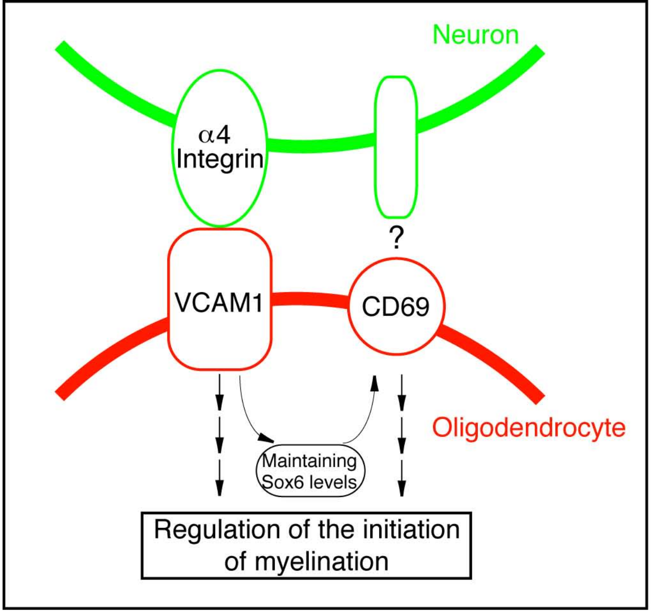

Supplementary Fig. 17. Proposed model for the role of the VCAM1 system in the regulation of myelination by oligodendrocytes. Oligodendrocyte VCAM1 positively regulates the initiation of myelination not only through the neuronal VCAM1 ligand α4 integrin but also through controlling the abundance of oligodendrocyte CD69. The ligand of CD69 in the CNS has not yet been identified.

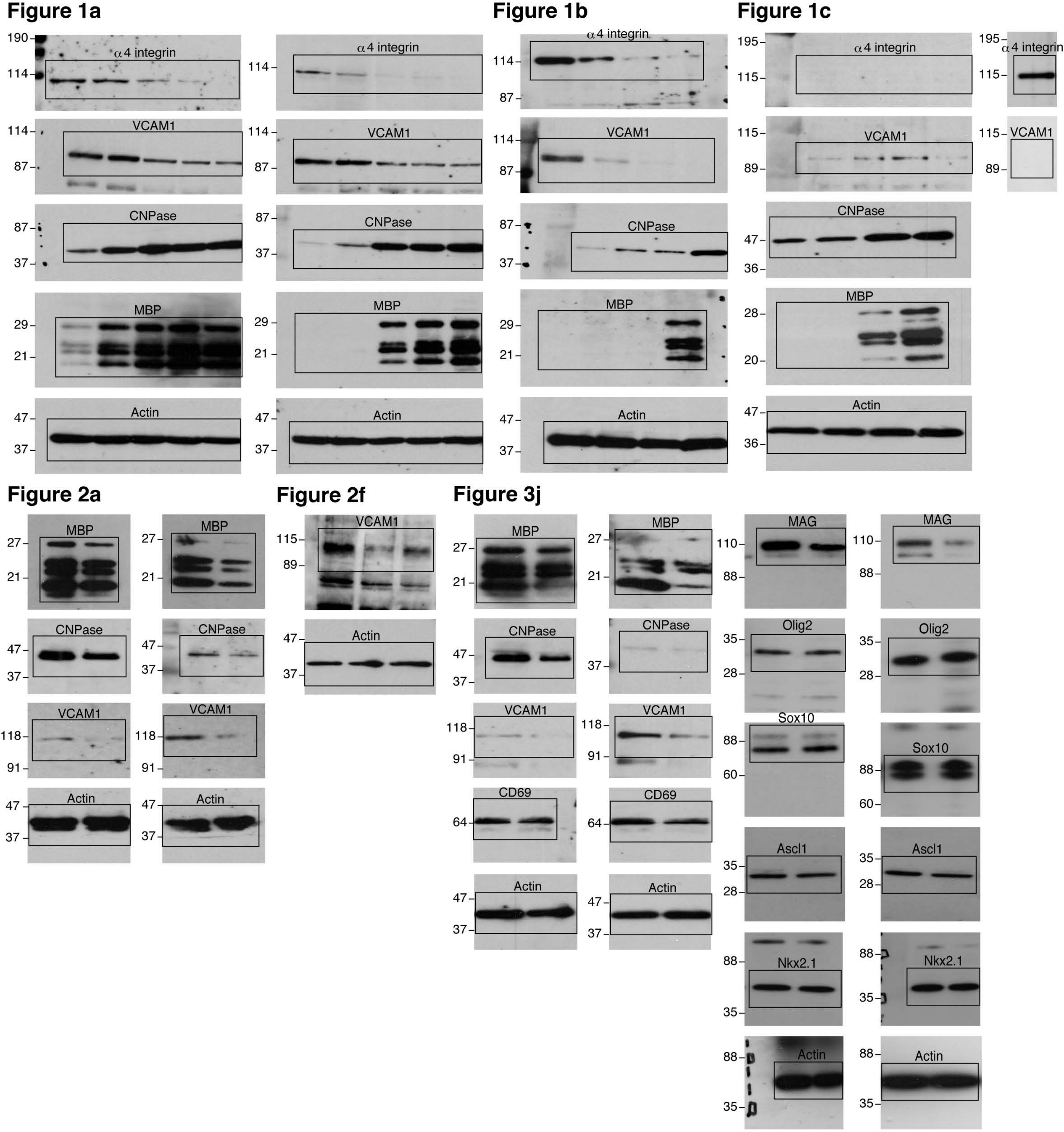

Supplementary Fig. 18. Original images of blots presented in the main paper.

Regions corresponding to cropped images are surrounded by black sequare lines.

Figure 5a

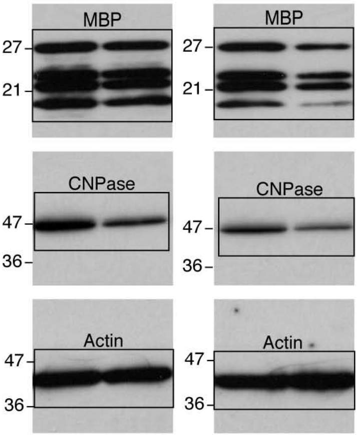

Figure 7a

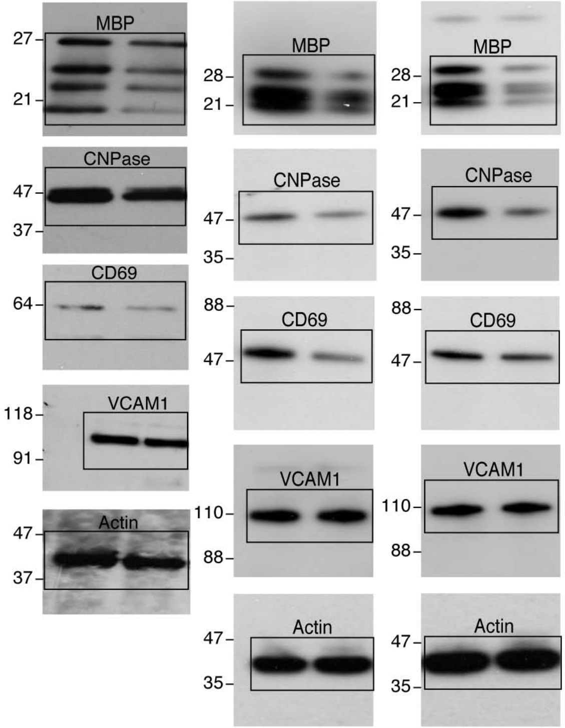

Supplementary Fig. 19. Original images of blots presented in the main paper.
Regions corresponding to cropped images are surrounded by black sequare lines.

Supplementary Table 1 Relative values of oligodendrocyte cell adhesion molecule transcripts.

Gene product name	Relative value	Standard deviation
Vcam1	100	11.7
Alcam1	23.6	4.58
Ncam1	3.99	0.995
Icam1	3.94	1.58

Microarray analyses for proliferating oligodendrocytes were performed using rat 3D-Gene oligo chips (n=3). Transcripts were extracted using cell adhesion molecule as the key word; their relative quantitative values are shown in descending order.

Supplementary Table 2 Oligodendrocyte transcripts were downregulated following VCAM1 knockdown.

Gene product name	Fold increase (log2 ratio)	*p*-Value
CD69	-1.27	0.002
Stefin A2	-1.19	0.005
Serglycin	-1.08	0.005
Interleukin-6	-0.98	0.014
Nitric oxide synthase	-0.97	0.021
Corticosteroid 11-β-dehydrogenase isozyme 2	-0.90	0.002
Colony stimulating factor 3	-0.89	0.014
Hemopexin	-0.86	0.009
Cxcl11	-0.81	0.014
Regulator of G-protein signaling 1	-0.81	0.002
Insulin-like growth factor-binding protein 3	-0.78	0.010
C11orf34 orthologue	-0.76	0.003
Ccl5	-0.75	0.021
Interferon-induced protein with TTR 2	-0.74	0.003
RNA helicase LGP2	-0.69	0.036
VCAM1	-0.32	0.001

Cells were treated with VCAM1 or control shRNA for 20 hours, and microarray analyses were performed using 3D-Gene oligo chips. The detected signals for the respective genes were normalized according to the global normalization method (n=3). Transcripts that were downregulated by VCAM1 knockdown, as well as VCAM1 transcripts, are shown in order. Since the knockdown was designed as a short-term experiment, the knockdown efficiency of VCAM1 was considered unlikely to be large; nevertheless, some initially downregulated transcripts associated with the knockdown have been identified.
